# Improving Photodynamic Therapy Anticancer Activity of a Mitochondria-Targeted Coumarin Photosensitizer Using a Polyurethane–Polyurea Hybrid Nanocarrier

**DOI:** 10.1021/acs.biomac.2c00361

**Published:** 2022-06-13

**Authors:** Joaquín Bonelli, Enrique Ortega-Forte, Anna Rovira, Manel Bosch, Oriol Torres, Cristina Cuscó, Josep Rocas, José Ruiz, Vicente Marchán

**Affiliations:** † Departament de Química Inorgànica i Orgànica, Secció de Química Orgànica, Institut de Biomedicina de la Universitat de Barcelona (IBUB), Universitat de Barcelona (UB), E-08028 Barcelona, Spain; ‡ Nanobiotechnological Polymers Division, Ecopol Tech, S.L., El Foix Business Park, Indústria 7, L’Arboç del Penedès, 43720 Tarragona, Spain; § Departamento de Química Inorgánica, Universidad de Murcia, Institute for Bio-Health Research of Murcia (IMIB-Arrixaca), E-30071 Murcia, Spain; ∥ Unitat de Microscòpia Òptica Avançada, Centres Científics i Tecnològics (CCiTUB), Universitat de Barcelona (UB), E-08028 Barcelona, Spain

## Abstract

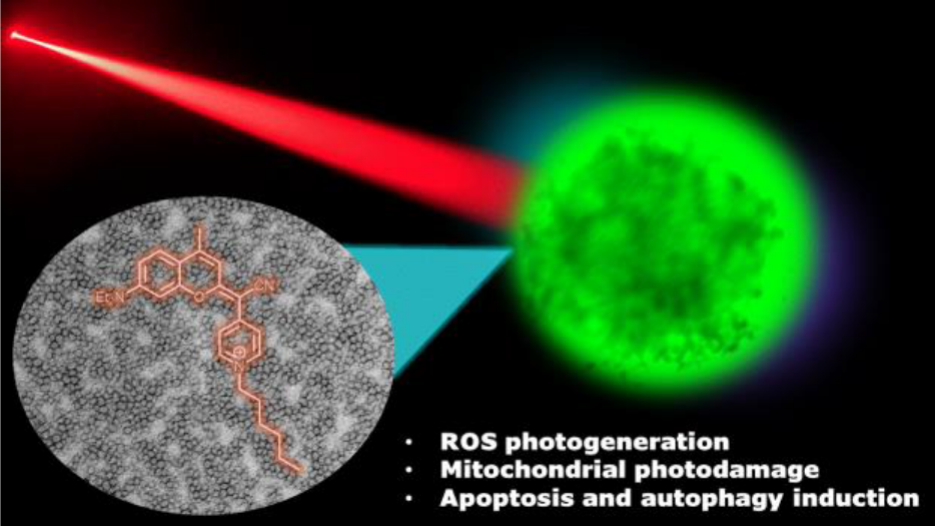

Integration of photosensitizers (PSs) within nanoscale delivery systems offers great potential for overcoming some of the “Achiles’ heels” of photodynamic therapy (PDT). Herein, we have encapsulated a mitochondria-targeted coumarin PS into amphoteric polyurethane–polyurea hybrid nanocapsules (NCs) with the aim of developing novel nanoPDT agents. The synthesis of coumarin-loaded NCs involved the nanoemulsification of a suitable prepolymer in the presence of a PS without needing external surfactants, and the resulting small nanoparticles showed improved photostability compared with the free compound. Nanoencapsulation reduced dark cytotoxicity of the coumarin PS and significantly improved in vitro photoactivity with red light toward cancer cells, which resulted in higher phototherapeutic indexes compared to free PS. Importantly, this nanoformulation impaired tumoral growth of clinically relevant three-dimensional multicellular tumor spheroids. Mitochondrial photodamage along with reactive oxygen species (ROS) photogeneration was found to trigger autophagy and apoptotic cell death of cancer cells.

## 1 Introduction

Fluorophores based on small organic molecules have become powerful tools in diagnosis, prognosis, and bioimaging applications, especially those operating in the far-red to near-infrared (NIR) region of the electromagnetic spectrum because the radiation of long wavelengths is nontoxic, exhibits minimal interference from tissue autofluorescence, and penetrates deeper into biological tissues.^1^ In addition, many organic fluorophores exhibit the ability to generate cytotoxic reactive oxygen species (ROS) in the presence of molecular oxygen and under certain excitation conditions, thus allowing their use as photosensitizers (PSs) in photodynamic therapy (PDT), which is an emerging clinically approved procedure for treating several cancers, including bladder, lung, skin, esophageal, brain, and ovarian cancers.^2^ PDT is also a well-established modality in dermatology, ophthalmology, dentistry, and cosmetics, as well as in other nonclinical fields (e.g., eradication of viruses and other pathogens).^3^ Hence, organic fluorophores exhibiting optimal physicochemical, photophysical, and photochemical properties are promising candidates for clinical phototheranostics because they provide in a single chemical entity optical imaging and photodynamic treatment of a given pathology.^4^

Despite the large number of compounds that have been described so far that can act as PSs, both porphyrinoids and nonporphyrinoids, most of them suffer from several drawbacks, and only a limited number of them have received approval for clinical use.^5^ Poor aqueous solubility, aggregation, low photostability, concentration-dependent toxicity, and rapid clearance by excretion organs hamper, in most of the cases, their transition to clinical acceptance. The ability of PSs to target cancer cells while sparing healthy cells, the O_2_-dependent nature of PDT, and the capacity of penetration of light required for activation in a given target tissue also determine the efficacy and clinical outcome of PDT agents, especially for combating hypoxic deep-seated tumors.^6^ Therefore, many efforts have been invested by researchers to overcome some of the “Achilles’ heels” of PDT by developing PSs based on alternative chemical entities with optimal physicochemical, photophysical, and photochemical properties, as well as with good biological performance. However, the difficulties associated with combining all of them in a single molecule demands to integrate known and de novo-synthesized PSs within nanoscale delivery systems. Besides protecting the PS from degradation and enabling specific accumulation in different tumor tissues, nanocarriers can strongly influence its photophysical properties^7^ and, consequently, there is an increased interest in the development of novel nanoPDT carriers.^8^ Inorganic nanoparticles,^9^ PEGylated dendrimers,^10^ liposomes,^11^ polymerosomes,^12^ and protein^13^ and polymeric nanoparticles^14^ have been investigated, among others, as organic fluorophores’ nanocarriers for bioimaging and PDT applications, as well as quantum dots being some of them PSs by themselves.^15^

Polyurethane-based polymers and copolymers^16^ are generally considered biocompatible products for medical applications because they have been used for producing, for example, catheters^17^ and stents.^18^ ECOSTRATAR technology^19^ has been recently introduced in nanomedical solutions to provide robust, nontoxic, and long-circulating polyurethane-polyurea hybrid nanocapsules (NCs) for the stabilization of hydrophobic compounds in aqueous media.^20^ Polyurethane chemistry also facilitates the incorporation of suitable functional groups and targeting ligands on the NCs’ surface for promoting preferential accumulation in specific locations.^21^ Because the reduction of pH values in specific areas has been mostly associated with some types of dysfunctions or abnormal biological situations such as in the location of atheroma plaques in damaged arteries,^22^ in inflamed zones of tissues micromilieu caused by immune system activation mechanisms,^23^ or in the solid tumor microenvironment (TME),^24^ the introduction of amphoteric groups on the NCs’ surface triggers accumulation at pH media below 7.2 by selective cationization of surface amino groups.^25^ This targeted encapsulation strategy opens the door to exploring the biological activity of hydrophobic drugs in different medical fields, tuning the NCs’ surface to modify their biological behavior.^26^ In this context, we have recently described polyurethane-polyurea hybrid NCs loaded with two cell impermeable cyclometalated Ir(III) complexes whose anticancer activity could be investigated, thanks to their nanoencapsulation.^27^ Such Ir(III)-loaded nanoparticles were found to be completely stable in complete human AB serum but degradable in the presence of glutathione owing to the incorporation of disulfide bonds in the polymeric wall. Moreover, in vivo safety and biodistribution assays have been carried out using this type of NCs by system injection through the tail vein, in order to elucidate associated toxicity and preferential accumulation in ectopic and orthotopic lung cancer tumors, respectively, yielding very good results in both models.^28^

**Figure 1:**
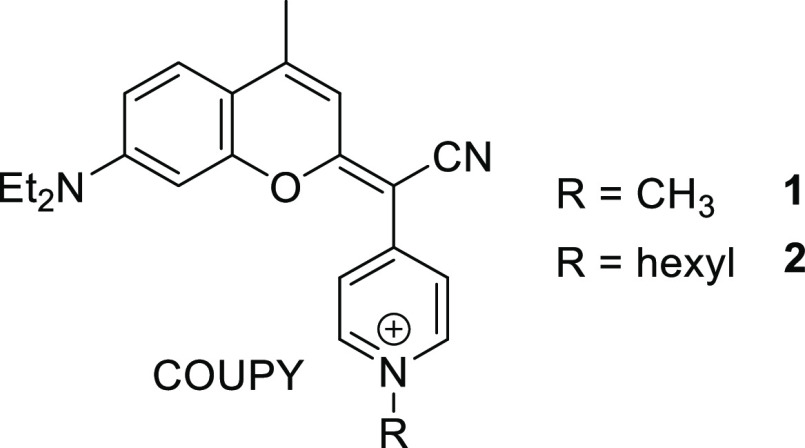
Structure of COUPY-based PSs investigated in this work.

Herein, we have explored the encapsulation of a new class of coumarin-based fluorophores (COUPYs) into NCs based on ECOSTRATAR technology with the aim of developing novel phototheranostic agents for nanoPDT applications. Besides being small and amenable to structural modifications, COUPY dyes exhibit attractive photophysical properties such as absorption and emission in the far-red/NIR region, large Stokes’ shifts, and brightness.^29^ In addition, COUPY derivatives are cell membrane-permeable in living cells and, depending on their structure, accumulate preferentially in the mitochondria owing to the presence of the lipophilic positively charged N-alkyl pyridinium moiety.^30^ Recently, we have investigated structure–activity relationships (SAR) within the COUPY scaffold and identified several PS candidates whose phototoxicity was related with ROS generation, even under hypoxia.^31^ Among them, COUPY derivatives **1** and **2** (Figure 1) were able to promote cell death both by apoptosis and autophagy induction after visible light irradiation and showed good phototherapeutic indexes. In this work, we have successfully encapsulated coumarin **2** in polyurethane-polyurea hybrid NCs and demonstrated that key parameters for bioimaging applications and photostability were significantly improved. Moreover, the PDT activity of COUPY **2**-loaded NCs was investigated in two-dimensional (2D) monolayer cancer cells as well as in clinically relevant three-dimensional (3D) multicellular tumor spheroids, and their mechanism of action was studied in detail.

## 2 Experimental Section

### 2.1 Photophysical Characterization

The ultraviolet–visible (UV–vis) absorption and emission spectra of coumarin **2** were recorded in ACN, EtOH, and H_2_O. Milli-Q water suspensions were used for **COUPY 2**-loaded NCs (**NC-COUPY-2**). Absorption spectra were recorded in a Jasco V-730 spectrophotometer at room temperature. Emission spectra were registered in a Photon Technology International (PTI) fluorimeter. Fluorescence quantum yields (Φ_F_) were measured using a comparative method using cresyl violet in ethanol (Φ_F; Ref_ = 0.54 ± 0.03) as the reference. Then, optically matched solutions of the compounds and cresyl violet were excited, and the fluorescence spectrum was recorded. The absorbance of sample and reference solutions was set below 0.1 at the excitation wavelength, and Φ_F_ values were calculated using the following eq 1:
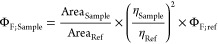
1where Area_Sample_ and Area_Ref_ are the integrated fluorescence for the sample and the reference, and η_Sample_ and η_Ref_ are the refractive index of sample and reference solutions, respectively. The uncertainty in the experimental value of Φ_F_ has been estimated to be approximately 10%.

Photostability of the free coumarin (**COUPY 2**) and of **COUPY 2**-loaded NCs (**NC-COUPY-2**) was investigated by monitoring fluorescence bleaching of a MilliQ water solution of the compounds at 37 °C irradiated with a high power 505 nm LED (100 mW/cm^2^). Fluorescence intensity values were recorded at *t* = 0 (*F*_0_) and after different irradiation times (*F*).

### 2.2 Singlet Oxygen Measurements

Singlet oxygen quantum yields of **COUPY 2** and **NC-COUPY-2** were determined in an air-saturated 1:1 (v/v) mixture of H_2_O and EtOH (bubbled for 15 min) using 1,3-diphenylisobenzofuran (DPBF) as a chemical trap upon green light irradiation using a high-power light-emitting diode (LED) source (505 nm, 100 mW cm^–2^) following previously reported procedures.^32^ Upon reaction with singlet oxygen, the fluorescent scavenger DPBF decomposes into a colorless product.^33^ The starting absorbance of DPBF in EtOH/H_2_O 1:1 was adjusted around 1.0 (50 μM); then, the compounds were added to the cuvette, and their absorbance was adjusted around 0.06 at the light irradiation wavelength (505 nm). Then, the decrease in the absorbance of DPBF at 411 nm was monitored. The linear relation of the variation in the absorbance (*A*_0_ – *A*_t_) of DPBF at 411 nm against irradiation time was plotted. Singlet oxygen quantum yields were calculated by the following eq 2:
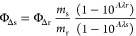
2where Φ_Δr_ is the reference singlet oxygen quantum yield of methylene blue (Φ_Δr_ = 0.52 in H_2_O),^34^*m* is the slope, and *A*λ*s* and *A*λ*r* are the absorbance of the compounds and of the reference (methylene blue, MB) at the irradiation wavelength, respectively. The slopes of MB, **COUPY 2**, and **NC-COUPY-2** were 0.10, 0.0040, and 0.0076, respectively.

### 2.3 Fluorescence Imaging by Confocal Microscopy

HeLa cells were maintained in DMEM (Dulbecco’s modified Eagle medium) containing high glucose (4.5 g/L) and were supplemented with 10% fetal bovine serum (FBS), 50 U/mL penicillin–streptomycin, and 2 mM l-glutamine. For cellular uptake experiments and posterior observation under the microscope, cells were seeded on glass bottom dishes (P35G-1.5-14-C, Mattek). Twenty-four hours after cell seeding, cells were incubated at 37 °C for 30 min with free and encapsulated coumarin (1 μM) in supplemented DMEM. To determine the internalization mechanism of both compounds, low-temperature incubations were performed at 4 °C during 30 min in the same biological medium and at the same concentration (1 μM). Then, cells were washed three times with DPBS (Dulbecco’s phosphate-buffered saline) to remove the excess of the compounds and kept in low glucose DMEM without phenol red supplemented with Hepes 10 mM for fluorescence imaging.

All microscopy observations were performed using a Zeiss LSM 880 confocal microscope equipped with a heating insert (P S1, Pecon). In the case of low-temperature internalization, cells were kept at RT. Cells were observed using a 63× 1.4 oil immersion objective. The compounds were excited using the 561 nm laser and detected from 570 to 670 nm. Image analysis was performed using Fiji.^35^ Unless otherwise stated, images are colorized using a Fire lookup table.

### 2.4 Biological Studies

Human cervix adenocarcinoma cell line, HeLa, and buffalo green monkey kidney cells, BGM, were cultured in DMEM supplemented with 10% FBS, 2 mM l-glutamine, 1% penicillin–streptomycin, and 1% nonessential amino acids. Human ovarian cisplatin-resistant cancer cells, A2780cis, were maintained in RPMI-1640 cell medium supplemented with 10% FBS, 2 mM l-glutamine, and 1% penicillin–streptomycin. Cisplatin acquired resistance was maintained by adding 1 μM of water-diluted cisplatin to cell culture flasks every second passage. All the cells were cultured in humidified incubators at 310 K in a 5% CO_2_ atmosphere, subcultured two or three times a week with appropriate densities, and were confirmed to be mycoplasma-free using a standard Hoechst DNA staining method.

#### 2.4.1 Photocytotoxicity Evaluation in 2D Monolayer Cells

HeLa cells were used to determine photocytotoxicity of the tested complexes. Cells were cultured in 96-well plates at a density of 5000 cells/well in complete medium and incubated for 24 h in normoxia (21% O_2_) or hypoxia (2% O_2_). A detailed setup for hypoxia experiments has been previously described.^31^ Serial dilutions of the compounds (final DMSO % below 0.4) or nanoparticles (water-diluted) were added at the final concentrations in the range of 0 to 200 μM in a final volume of 100 μL per well. The treatment schedule was performed as follows: 0.5 h incubation in the dark followed by 1 h incubation under irradiation conditions by placing the photoreactor EXPO-LED from LuzChem (Canada) fitted with white lamps (final light intensity applied of 3 mW/cm^2^ at λ_max_ = 520 nm; 2.6 mW/cm^2^ at λ_max_ = 595 nm) inside the CO_2_ incubator. Alternatively, LuzChem well plate illuminator fitted with red lamps (89 mW/cm^2^ at λ_max_ = 630 nm) was used for 0.5 h or 1 h. Control samples were kept in dark conditions during the phototoxic schedule in a humidified CO_2_ incubator. Then, 48 h treatment-free cell recovery period was allowed; temperature throughout the experiment was maintained at 310 K. Cell medium was aspirated by suction, cells washed with saline PBS buffer, and loaded with 50 μL of MTT solution (1 mg/mL) for additional 4 h, then removed, and 50 μL of DMSO was added to solubilize the purple formazan crystals formed in active cells. The absorbance was measured at 570 nm using a microplate reader (FLUOstar Omega), and the IC_50_ values were calculated based on the inhibitory rate curves using the next eq 3:
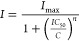
3where *I* represents the percentage inhibition of viability observed, *I*_max_ is the maximal inhibitory effect, *IC*_50_ is the concentration that inhibits 50% of maximal growth, *C* is the concentration of the treatment, and *n* is the slope of the semi-logarithmic dose–response sigmoidal curves. The nonlinear fitting was performed using SigmaPlot 14.0 software. All experiments were performed in three independent studies with triplicate points per concentration level (*n* = 3 biologically independent replicates).

#### 2.4.2 Photocytotoxicity Evaluation on 3D Multicellular Spheroids

For the generation of HeLa multicellular tumor spheroids (MTCS), 96-well Corning microplates with ultralow attachment surface coating were used. Briefly, a single suspension of HeLa cells at a density of 5 × 10^3^ cells/well was prepared in complete DMEM medium and dispensed into wells. The plates were covered and transferred to incubator at 310 K with 5% CO_2_ atmosphere. Within 3 days, uniform 200 μm diameter MTCS were formed from cell suspension and were maintained under these conditions. At day 3, MTCS were incubated with tested agents (2 μM) for 6 h and then irradiated with red light for 0.5 h. Treatments were then replaced with fresh cell media and changed every 3 days by replacing 50% of the media. The formation, integrity, diameter, and volume of the multicellular tumor spheroids (MCTS) were monitored using a DMi1 inverted phase contrast microscope (Leica Microsystems) over a span of 9 days.

#### 2.4.3 ROS Generation

ROS levels were determined using the 2′-7′dichlorofluorescein diacetate (DCFH-DA). HeLa cells were seeded onto 96-well plates at 2 × 10^4^cells/well for 24 h in a humidified CO_2_ incubator. Alternatively, MCTS were cultured in ULA 96-well plates and spheroids were formed within 3 days. Then, cells were stained with 10 μM of DCFH-DA for 0.5 h and washed with PBS prior treatments. Tested compounds were then administered in cell media for the allowed time, and visible light irradiation was then applied for 1 h. Cells were then washed with PBS twice and imaged using a Zeiss Axio microscope with the 40× objective using the green fluorescence channel and the intensities analyzed with ImageJ software. The assay was performed in three independent experiences (*n* = 3 per replicate). Alternatively, ROS generation was analyzed by flow cytometry following a similar procedure. Briefly, HeLa cells were seeded onto 12-well plate (2 × 10^5^ cells/well). Treatments with tested agents for 1 h were applied. Cells were trypsinized, and pellets were resuspended in DCFH-DA staining solution for 30 min. Samples were then irradiated for 1 h and subjected to flow cytometry (FACSCAlibur BecktonDickinson; 10^4^ events acquired per sample), using λ_exc_ = 488 nm and λ_em_ = 530 ± 30 nm in the FL1-H channel. Three independent experiments were performed (*n* = 2 replicates).

#### 2.4.4 Mitochondrial Membrane Potential Assessment

Mitochondrial membrane potential (MMP) was evaluated with the fluorescent probe JC-1 chloride (Promocell). Briefly, HeLa cells in the density of 1.5 × 10^5^ were seeded for 24 h in complete medium on 12-well plates, and then treated with indicated concentrations of tested compounds for 0.5 h. Visible light irradiation was applied for 1 h (3 mW/cm^2^ at λ_max_ = 520 nm) using photoreactor EXPO-LED (Luzchem). Dark analogues were kept in the dark for 1.5 h. Untreated cells were used as a negative control, whereas CCCP (50 μM; 24 h) was used as a positive control for mitochondrial dysfunction. After drug exposure, treatment-containing media were removed, and cells were incubated with fresh media for 24 h. Then, staining JC-1 dye (1 μM) for 20 min was applied, and cells were subjected to flow cytometry (FACSCAlibur BecktonDickinson; 10^4^ events acquired per sample), using λ_exc_ = 488 nm, λ_em_ = 530 ± 30 nm (green), and 585 ± 30 nm (red) parameters to discriminate green JC1 monomers (FL1-H channel) and red JC1 aggregates (FL2-H channel). Three independent experiments were performed (*n* = 2 replicates).

#### 2.4.5 Apoptosis Induction

Cell death induction was evaluated using standard Annexin V-FITC staining. Briefly, HeLa cells were seeded in 12-well plates at a density of 1.5 × 10^5^cells/well and incubated overnight. Compounds and cisplatin (20 μM) were added following the described treatment schedule (0.5 h incubation +1 h irradiation) at IC_50_^LIGHT^ concentrations. Dark analogues were kept in the dark for 1.5 h. After 24 h of drug-free recovery period, cells were harvested by trypsinization, washed with PBS, centrifuged, and the pellets were resuspended in 200 μL of binding buffer. Then, Annexin V-FITC was added as instructed by the manufacturer (eBioscience). The resuspended cell solution was left at room temperature in the dark for 15 min prior to analysis by flow cytometry (FACSCalibur BecktonDickinson; 10^4^ events acquired per sample) with λ_exc_ = 488 nm using FL1 channels. Data were analyzed using FlowingSoftware version 2.5.1. The assay was performed in three independent experiences (*n* = 2 replicates).

#### 2.4.6 Autophagy Detection

Autophagic processes were detected using the fluorescent probe monodansylcadaverine (MDC; Sigma), as previously described.^36^ Briefly, HeLa cells at a density of 15,000 cells/cm^2^ were seeded onto confocal 8 μ-slide chambers (Ibidi) and allowed to attach and grow inside the CO_2_ incubator. Cells were then treated with equitoxic concentrations (close to IC_50_^LIGHT^) of tested compounds, following described phototoxicity schedules. Resveratrol (50 μM, 2 h) was used as a positive control.^37^ After irradiation, drug-containing media was replaced by fresh media, and a 6 h recovery period was allowed. Cells were then washed with PBS, stained with the selective autophagy marker MDC (50 μM in PBS) for 10 min at 310 K, washed again with PBS three times, and imaged under confocal microscopy (SP8 Leica systems, λ_exc_ = 405 nm). The number of MDC vesicles were counted and processed using ImageJ software.

#### 2.4.7 Cell Metabolism Measurements

The mitochondrial OXPHOS and glycolysis function of HeLa cells was measured by determining the oxygen consumption rate (OCR) and extracellular acidification rate (ECAR) with a Seahorse XFe96 extracellular flux analyzer. In brief, HeLa cells were seeded at a density of 3 × 10^4^ cells/well to the XFe96-well culture microplates (Seahorse Agilent) the day before. The sensor cartridge was hydrated through immersion on calibration buffer at 310 K in a non-CO_2_ incubator overnight. Buffered DMEM (Seahorse Bioscience) was used for the assay. Cells were treated for 2 h at indicated concentrations with testing compounds. Cellular metabolism was assessed using a XF Glycolytic Rate Test Kit. OCR and ECAR measurements were monitored in real time, and respiration rates were averaged before and after the injection of a mixture of complex III electron transport chain inhibitors (Rotenone/Antimycin A, 1 μM) to impair OXPHOS and glycolysis inhibitor (2-deoxyglucose, 50 mM) to block glucose metabolism. All tests had four replicates.

#### 2.4.8 Cell Cycle Distribution

Determination of the cell cycle distribution of HeLa cells was performed using a standard propidium iodide staining method. Briefly, HeLa cells were seeded onto 12-well plates at a density of 1.5 × 10^5^cells/well and incubated overnight. Compounds and cisplatin (20 μM) were added following the described treatment schedule (0.5 h incubation +1 h irradiation) at IC_50_^LIGHT^ concentrations. Dark analogues were kept in the dark for 1.5 h. After 24 h of the cell recovery period, cells were harvested by trypsinization and permeabilized in 70% ethanol for 1 h. Cells were then centrifuged and stained with propidium iodide for 30 min prior to analysis by flow cytometry (FACSCalibur BecktonDickinson; 10^4^ events acquired per sample) with λ_exc_ = 488 nm using an FL2-A channel. Data were analyzed using FlowingSoftware version 2.5.1. The assay was performed in three independent experiences (*n* = 2 replicates).

#### 2.4.9 Statistical Methods

All biological experiments were repeated at least in triplicate. Statistical analysis was performed using either analysis of variance (ANOVA) or unpaired t-test in GraphPad Prism software. P-values less than 0.05 were considered to be statistically significant.

## 3 Results and Discussion

### 3.1 Synthesis and Characterization of COUPY-Loaded NCs

**Scheme 1:**
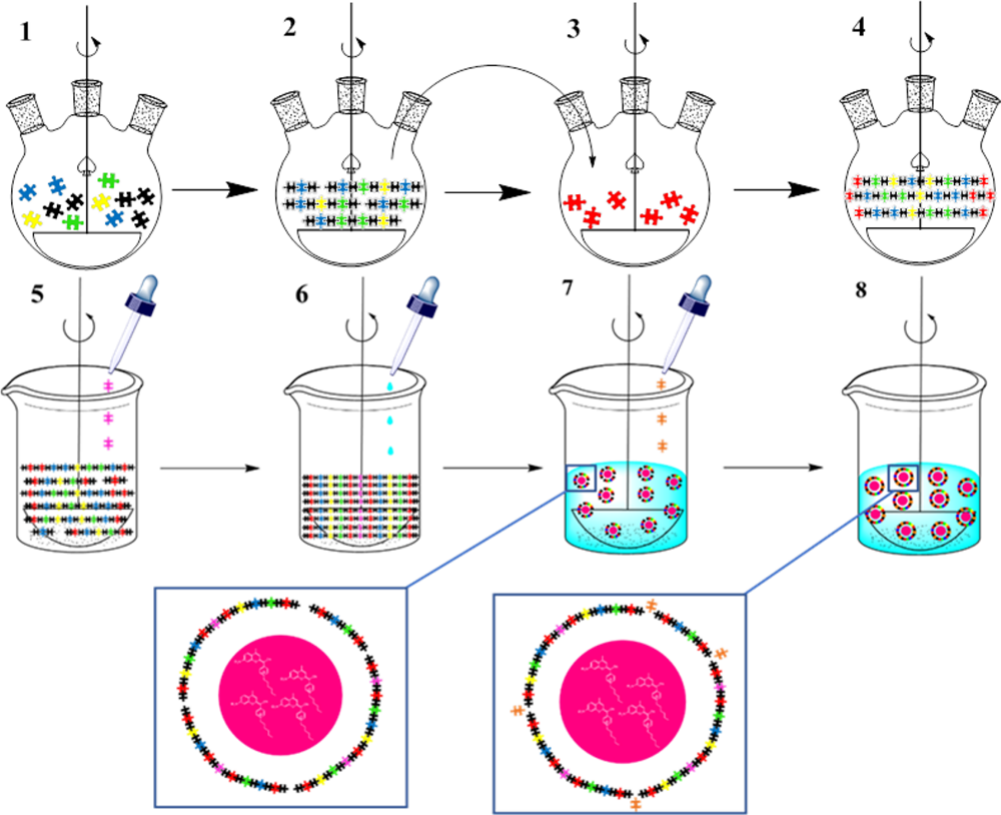
Schematic Representation of the Synthesis of the Amphiphilic Polyurethane–Polyurea Prepolymer (Steps 1–4) Followed the Nanoemulsion and Nanoencapsulation Processes (Steps 5–8) Puzzle pieces codes: black for isophorone diisocyanate; blue for YMER N-120; green for *N*-(3-dimethylaminopropyl)-*N*,*N*′-diisopropanolamine; yellow for 2,2′-dihydroxyethyl disulfide; red for 1,3-diamino-*N*-octadecylpropane; pink for l-lysine, and orange for diethylenetriamine.

The synthesis of COUPY-loaded NCs involves two main processes, as described in detail in the Supporting Information: (i) the preparation of a bifunctional NH_2_-terminal redox-responsive amphiphilic polyurethane–polyurea prepolymer and (ii) the fluorophore nanoencapsulation. As shown in Scheme 1, three different diol monomers (blue, yellow, and green pieces) were reacted first in the presence of an excess of isophorone diisocyanate (black pieces) (step 1) to furnish an NCO-terminated polyurethane polymer, as confirmed by Fourier transform infrared (FT-IR) analysis (step 2). Once the urethane stretching band growth reached a plateau, the product was dissolved in tetrahydrofuran (THF) and added over an excess of a hydrophobic diamine (red pieces) (step 3), which furnished the final NH_2_-capped polyurethane–polyurea prepolymer (step 4).

The amino functionalization allows the prepolymer storage, avoiding degradation of isocyanate groups by moisture. This self-emulsifiable prepolymer is the starting material for initiating the nanoencapsulation process (Scheme 1). First, the prepolymer was reactivated by the addition of an excess of isophorone diisocyanate (step not shown) and, after NCO bond appearance was confirmed by FT-IR, it was mixed with the COUPY PS (fuchsia circles in Scheme 1). Once coumarin was completely dissolved in the THF solution of the activated prepolymer, the dropwise addition of an aqueous solution of l-lysine (pink pieces) was started to extend the prepolymer chain, also furnishing an amphoteric polymer (step 5). Then, MilliQ water was added dropwise to form an inverted phase nanoemulsion (step 6), where the COUPY derivative was contained into the liposoluble core. Once oil in water nanoemulsion was defined, a polyamine (orange pieces) was added as a cross-linking agent to react with terminal NCO groups (step 7), providing robustness and resulting in the final NC formation (step 8). After 24 h of dialysis purification using a molecular porous membrane tubing with a 12–14 kDa MWCO, physicochemical and encapsulation yielding parameters of the resulting coumarin-loaded NCs were evaluated.

It is worth noting that all the chemical reactions performed during the encapsulation process (see steps 5–8 in Scheme 1) are carried out at the interphase of the emulsion, furnishing a hybrid, and ordered, polyurethane–polyurea wall where the hydrophilic groups face the external aqueous phase and lipophilic ones are internally (core)-oriented. As a consequence, this synthetic methodology would allow, if required, the NCs’ size, surface charge, and/or wall thickness to be easily modified by changing the ratio of monomers or the global amount of polymers because the self-emulsifiable prepolymer both drives nanodispersion stabilization and, after the final cross-linking, the generation of the NC.

**Figure 2:**
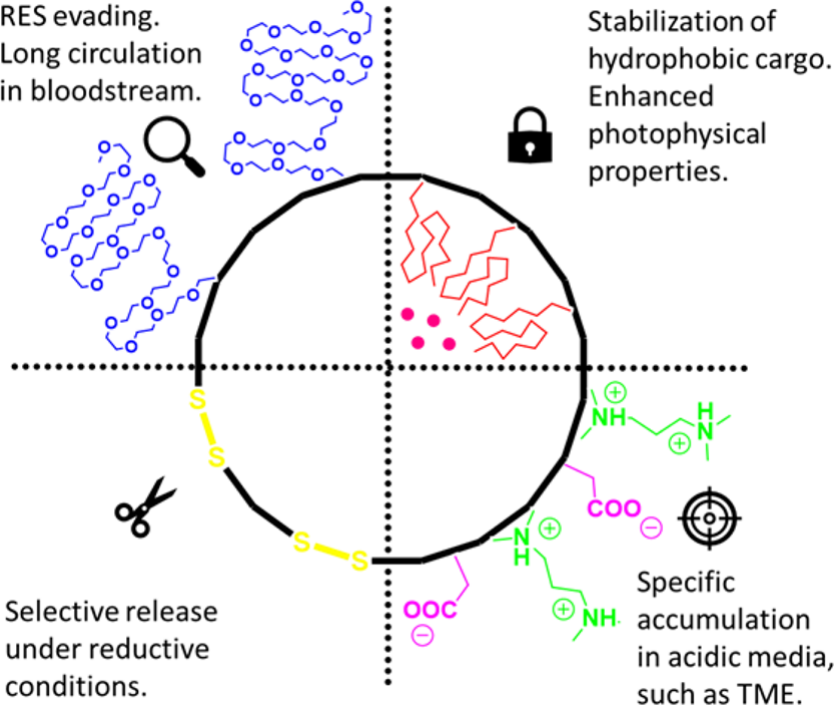
Schematic representation of the different moieties incorporated in the polyurethane-polyurea backbone of the NCs’ shell structure.

As illustrated in Figure 2, the polyurethane–polyurea backbone of the NCs’ shell incorporates moieties that enable distinctive and genuine performance, making the NCs sensitive to biological media variations. On the one hand, the incorporation of polyethylenglycol (PEG) chains ensures a long circulation lifetime in bloodstream and minimizes the clearance using the reticuloendothelial system (RES),^38^ while ionomeric groups facilitate accumulation in an acidic TME. On the other hand, core-oriented hydrophobic chains are expected not only to solubilize and stabilize the lipophilic cargo but also to positively influence its photophysical properties by providing a protective and nonpolar environment. Finally, NCs might be degraded under reductive conditions owing to the incorporation of disulfide bonds in the polymer backbone, which will facilitate the release of the PS.^25^

Following the general procedure described above, the encapsulation of coumarins **1** and **2** (Figure 1) was investigated. Strikingly, water acquired a pink color during dialysis of **COUPY 1**-loaded NCs (Figure S1), which indicated that the coumarin might have been released partially from the NCs. By contrast, no color was observed in water during purification of NCs synthesized with **COUPY 2** (Figure S2). Based on these observations, the amount of coumarins **1** and **2** inside NCs was quantified by UV–vis spectroscopy. As shown in Table S2, the encapsulation efficiency was very high for coumarin **2** (ca. 91%), and a high dye loading was reached (1.16 ± 0.01 mM) for **COUPY 2**-loaded NCs (**NC-COUPY 2**) considering that no surfactants had been used during the encapsulation process. However, consistent with the observations during dialysis purification, **COUPY 1**-loaded NCs (**NC-COUPY 1**) did not contain the expected dye, which indicates that the incorporation of the hexyl group in the coumarin moiety of the COUPY scaffold is required for the retention of the compound inside the hydrophobic environment provided by the NCs.

**Figure 3:**
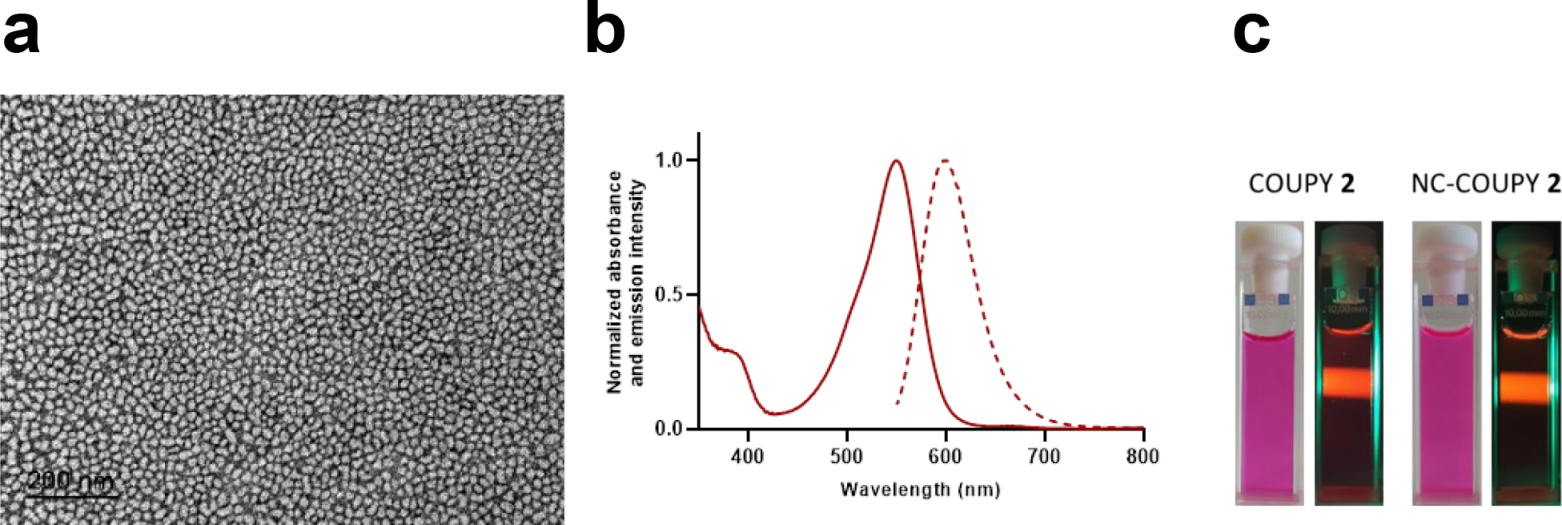
Characterization of **NC-COUPY-2**. (a) TEM micrograph (left). (b) UV–vis and emission spectra in water solution. (c) Photographic images of free and encapsulated **COUPY 2** in daylight and in the dark upon irradiation with a green LED source.

The size and morphology of **NC-COUPY 2** was then studied by dynamic light scattering (DLS) and by transmission electron microscopy (TEM), respectively. As shown in Figure S7, the average particle size distribution was centered approximately at 14.55 ± 0.53 nm (Table S3), and TEM micrographs revealed a roughly round shape and a homogeneous particle size (Figure 3). Other TEM micrographs of **COUPY 2**-loaded NCs are shown in Figure S8. As shown in Figure S9, the morphology of the NCs was also analyzed by high-resolution TEM (HR-TEM). Although nanocarriers are usually designed to facilitate accumulation at the tumor site by the enhanced and permeability and retention effect (EPR),^39^ smaller nanomedicines (e.g., 15–20 nm) are ideal for cancer therapy because of their superior tumor penetration.^40^ In addition, the degradability of the NCs in glutathione-supplemented PBS buffer (10 mM) was also investigated with the aim of reproducing the situation in the intracellular media of cancer cells, where the concentration of the reduced form of this tripeptide is about 10 times higher than that in normal cells. As expected, the release of the coumarin PS from **NC-COUPY 2** was confirmed after incubation in PBS supplemented with glutathione for 24 and 48 h at 37 °C (Figure S10), which suggests that the degradation of the nanoparticles and release of the PS could be triggered in cancer cells through the glutathione-mediated reduction of the disulfide bonds incorporated along the polyurethane backbone of the NC wall. The results from these experiments are in good agreement with our previous observations by TEM, which demonstrated that NCs loaded with iridium(III) complexes were selectively degraded in the presence of glutathione, while they remained completely stable after incubation at 37 °C in PBS and in serum AB.^27^

The Z-potential of **NC-COUPY 2** at three pH values was also measured to evaluate the pH-dependent amphoteric properties of the polymeric shell (Figure S11). As expected, the NCs were found to be slightly anionic at physiological pH (7.4) but become cationic entities at low pH values. Based on the sub-100 nm size and the pH-dependent properties, we would expect that this novel nanoplatform will be presumably benefited from both EPR effect and acidic TME to preferentially target the tumor tissue in vivo. Regarding to its biodistribution, it is worth considering the long circulation times in the blood stream of small size nanoparticles (∼12 nm) and their superior flux into tumors, which would lead to favorable toxicity profiles in vivo.^40^ In addition, the intrinsic fluorescence of the COUPY cargo along with the homogenous particle size could facilitate biodistribution and pharmacokinetic studies as well as noninvasive imaging of **NC-COUPY 2** in vivo.

### 3.2 Photophysical and Photochemical Characterization of COUPY-Loaded NCs

Having at hand **COUPY 2**-loaded NCs, we investigated the effect of encapsulation on the spectroscopic and photophysical properties of the coumarin fluorophore (absorption and emission spectra, as well as fluorescence quantum yield (Φ_F_)). Considering that the NCs are dispersed in H_2_O but that the environment around the cargo is hydrophobic, the photophysical properties of the coumarin alone were also studied in three solvents of different polarities (H_2_O, ethanol, and ACN) for comparison purposes. The UV–vis absorption and emission spectra are shown in Figure 3 (**NC-COUPY 2**) and S12 (COUPY **2**), and the photophysical properties are summarized in Table S4. As shown in Figure 3, aqueous solutions of **COUPY 2**-loaded NCs showed a deep pink color owing to an intense absorption band in the yellow-red region of the electromagnetic spectrum with an absorption maximum centered at 550 nm. Interestingly, the absorption maximum of the encapsulated coumarin was slightly redshifted (ca. 5 nm) with respect to that of the free compound in H_2_O (λ_abs_ = 545 nm for **COUPY 2**). The fact that the absorption maximum value for **NC-COUPY 2** was similar to that of the free coumarin in ACN (λ_abs_ = 550 nm) and EtOH (λ_abs_ = 554 nm) accounts for the hydrophobic and protective environment inside the NCs. By contrast, the emission of the coumarin, which was located in the far-red to NIR region, was less sensitive to the polarity of the environment, and similar emission maxima wavelengths were obtained both for the encapsulated (λ_em_ = 600 nm) and free coumarin (λ_em_ = 602–604 nm depending on the solvent). As shown in Table S4, the fluorescence quantum yield for **NC-COUPY 2** was higher than that of the free coumarin in H_2_O (Φ_F_ = 0.36 and 0.20, respectively), which again can be attributed to the hydrophobicity around the fluorophore inside the NCs.

**Figure 4:**
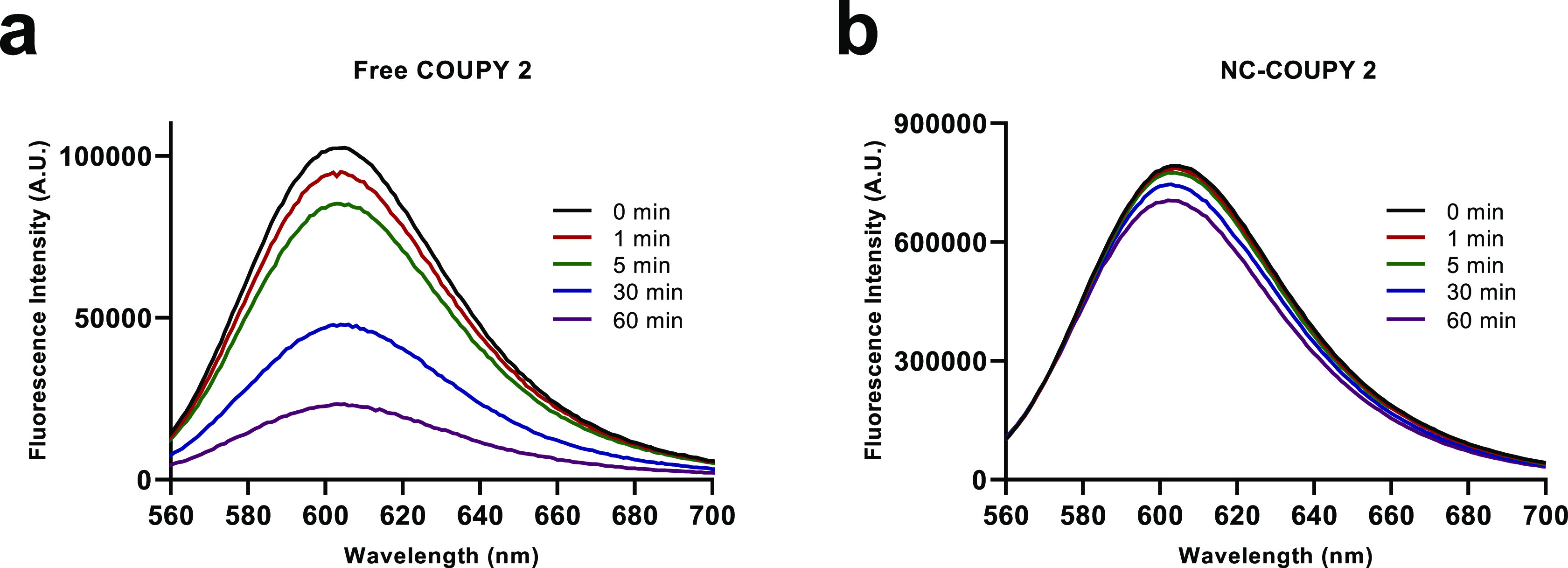
Emission spectra of **COUPY 2** (a) and **NC-COUPY-2** (b) after green LED irradiation at different times.

The photostability of **COUPY 2**, either alone or encapsulated, was also investigated in PBS under green light irradiation. To our delight, encapsulation had a clear positive effect on the photostability of the fluorophore, which was much higher than that of the free coumarin. As shown in Figure 4 and S13, **NC-COUPY 2** were found highly photostable up to light fluences larger 400 J cm^–2^, which are more than 20-fold higher than those used in bioimaging experiments with living cells. In summary, all these observations allowed us to conclude that the encapsulation of COUPY-based PSs in polyurethane–polyurea hybrid NCs had a positive effect in key photophysical properties for bioimaging applications because the hydrophobic environment around the organic fluorophore led to an improvement of its fluorescence emission yield and photostability, as well slightly red-shifting the maximum absorption.

Furthermore, the singlet oxygen generation by **NC-COUPY 2** was investigated by using 1,3-diphenylisobenzofuran (DPBF) as a ^1^O_2_ scavenger and methylene blue (MB) as a reference in air-saturated EtOH/H_2_O 1:1 (v/v) and compared with that of the free coumarin **2**. As shown in Figures S14 and S15, a gradual decrease in the absorbance of DPBF at 411 nm was observed upon irradiation with green light in the presence of the compounds, thereby confirming the generation of singlet oxygen. The fact that this process was slightly more efficient when the coumarin was encapsulated (Φ_Δ_ = 0.04 for **NC-COUPY 2** vs Φ_Δ_ = 0.02 for **COUPY 2**) suggests that nanoencapsulation in a hydrophobic environment has a positive effect on type II PDT photochemical reactions, leading to the generation of singlet oxygen. This conclusion is supported by the fact that the singlet oxygen production for the free coumarin **2** was much more efficient in DCM (Φ_Δ_ = 0.11)^31^ than in EtOH/H_2_O 1:1 (v/v) (Φ_Δ_ = 0.02).

### 3.3 Fluorescence Imaging of NC-COUPY 2 in Living Cells

**Figure 5:**
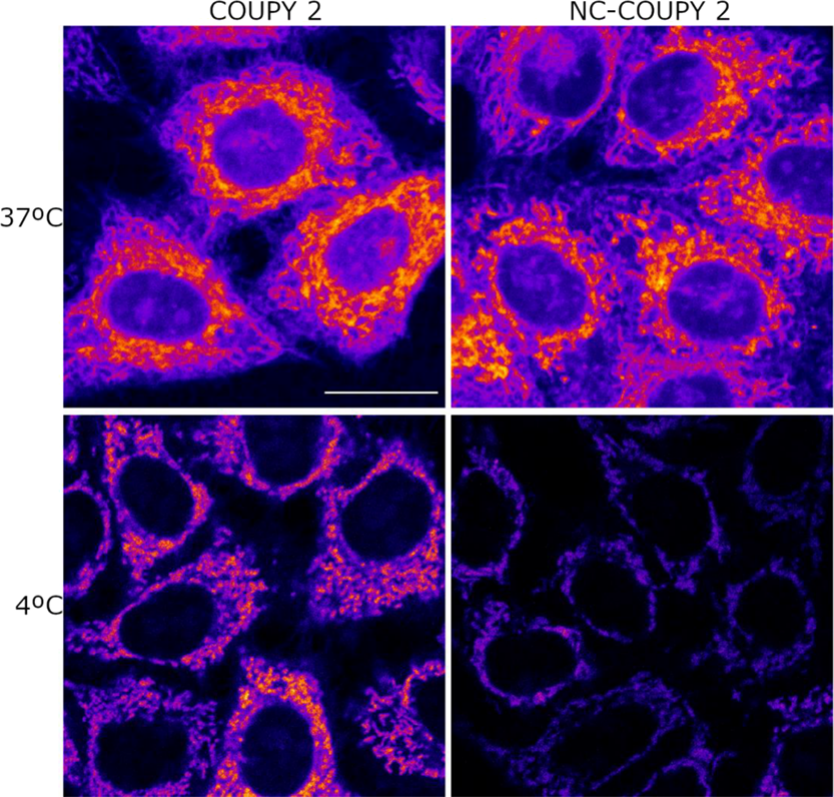
Cellular uptake of **COUPY 2** and **NC-COUPY 2** at 37 and 4 °C. Single confocal planes of HeLa cells incubated with the compounds at 1 μM for 30 min at 37 °C or 4 °C. Scale bar: 20 μm.

The cellular uptake of **COUPY 2**-loaded NCs was investigated in living HeLa cells by confocal microscopy and compared with that of the free coumarin with the aim of assessing the effect of encapsulation on the internalization of the PS. As shown in Figure 5, the fluorescence signal after incubation with **NC-COUPY 2** (1 μM, 30 min, 37 °C) and irradiation with a yellow light laser (λ_ex_ = 561 nm) was clearly observed inside the cells, mainly in mitochondria, which suggested that the NCs were able to cross the cell membrane, even after shorter incubations times (Figure S16). Strikingly, this pattern of staining was similar to that obtained for the free coumarin (Figure 5), which might be attributed to the fact that the NCs liberate very quickly the cargo coumarin once internalized and, for this reason, accumulation in the coumarin final target organelles was observed. As previously stated, glutathione-mediated reduction of the disulfide bonds incorporated in the polymeric wall of the NCs might account for the rapid release of the coumarin cargo, which can be explained by the high concentration of this tripeptide and other reducing biomolecules in cancer cells compared with normal cells.^27,41^ These observations were supported by the measurement of the mean fluorescence intensities for the mitochondria, nucleoli, and cytoplasm, which were quite similar both for the **COUPY 2**-loaded NCs and for the free coumarin (Figure S17). In addition, colocalization experiments with mitotracker green (MTG) (Figure S18) led to the same Pearson’s coefficients for **COUPY 2** (0.95) and **NC-COUPY-2** (0.94), which confirmed a perfect correlation between the coumarin signal and that of MTG. Similarly, Manders’ coefficients were quite high in both compounds (*M*1, *M*2 = 0.89 for **COUPY 2**; *M*1 = 0.83, *M*2 = 0.95 for **NC-COUPY-2**). As previously found with **COUPY 2** alone,^31^ the mitochondria of HeLa cells after incubation with **NC-COUPY-2** showed a characteristic donut-shaped morphology after excitation with the laser beam of the microscope (Figure S19), which point out to the mitochondria stress and could be related with ROS generation upon light irradiation.^42^

To further investigate the cellular uptake of **COUPY 2**-loaded NCs, low-temperature incubation experiments were also carried out. As shown in Figure 5, the intensity of the overall fluorescence signal was clearly reduced at 4 °C in the case of **NC-COUPY 2** (Figure S20), thereby suggesting that the nanoencapsulated form requires an enabled active transport to be internalized. This result is in good agreement with previous cellular uptake studies with Ir(III)-loaded NCs by inductively coupled plasma-mass spectroscopy (ICP-MS) that demonstrated that energy-dependent mechanisms are involved in the internalization of small polyurethane-polyurea hybrid NCs.^25^

### 3.4 Biological Activity of NC-COUPY 2

#### 3.4.1 Phototoxic Activity Determination in 2D Monolayer Cells

The efficacy of **NC-COUPY 2** as a nanoPDT agent was evaluated under irradiation with monochromatic red light (89 mW/cm^2^ at λ_max_ = 630 nm) and with broadband visible light (3 mW/cm^2^ at λ_max_ = 520 nm; 2.6 mW/cm^2^ at λ_max_ = 595 nm). Normoxic (21% O_2_) and hypoxic conditions (2% O_2_) were set up to investigate photodynamic effects under challenging low-oxygen environments. The antiproliferative activity of the nanoformulation **NC-COUPY 2** in the dark (dark cytotoxicity) and under light irradiation (phototoxicity) was evaluated in cervix adenocarcinoma cells (HeLa), cisplatin-resistance ovarian cancer cells (A2780cis), and nontumorigenic renal cells (BGM), and the results were compared with those of the free compound **COUPY 2** to evaluate the effect of nanoencapsulation. The parent compound **COUPY 1** was also included for comparison.

**Figure 6:**
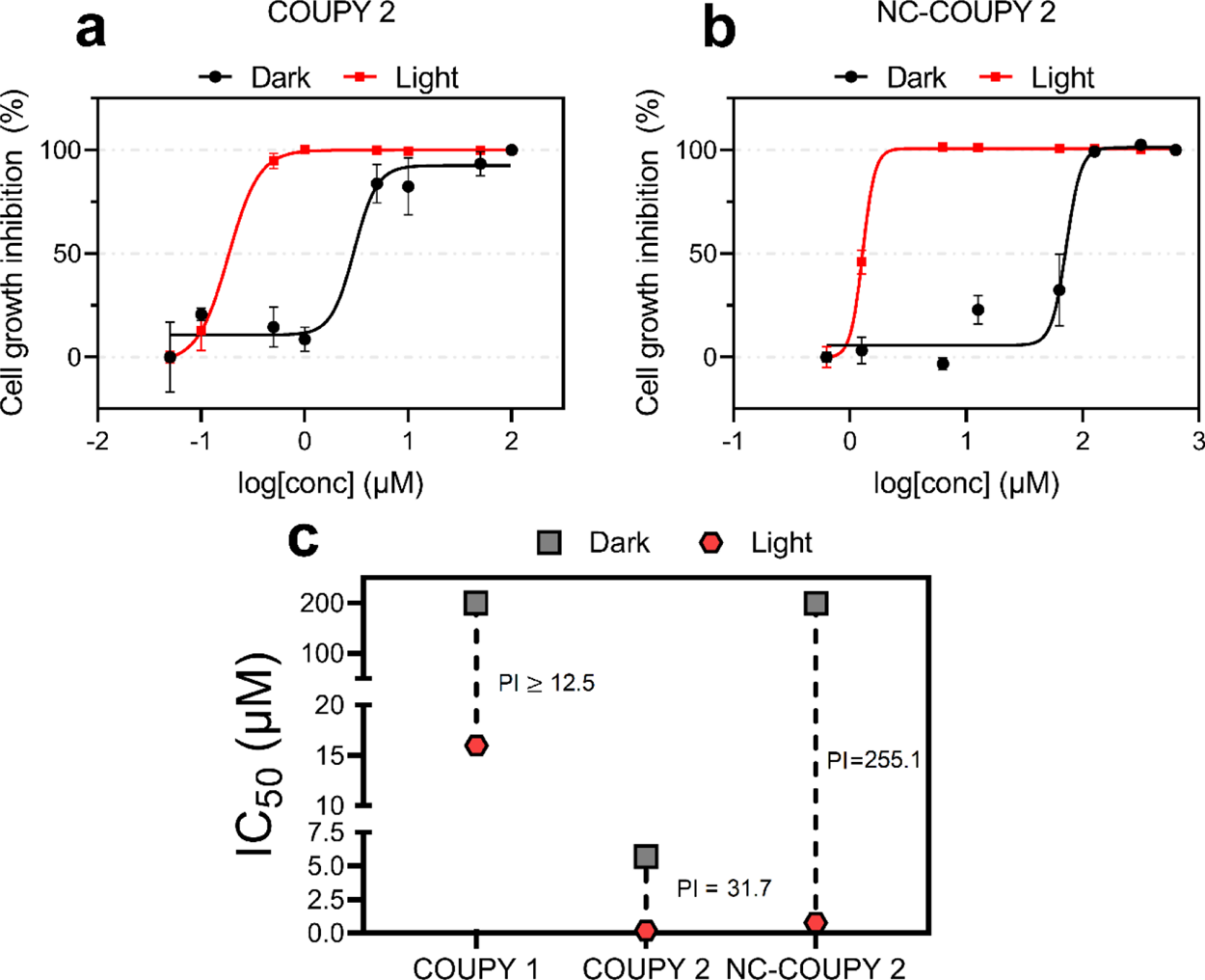
Dose–response curves of **COUPY 2** (a) and **NC-COUPY 2** (b) in HeLa cells. (c) Comparison of half-maximal inhibitory concentration (IC_50_) and phototoxic index (PI) values for light-activated COUPY compounds (0.5 h in dark +1 h red light irradiation followed by 48 h drug-free recovery period) in HeLa cells.

As already reported in our previous work,^31^ a dramatic increase in dark cytotoxicity was observed for coumarin **2** treatment (IC_50_^DARK^ = 5.7–5.9 μM) in contrast to coumarin **1** (IC_50_^DARK^ > 200 μM), which is ascribable to the *N*-alkylation of the pyridine moiety in the COUPY scaffold with the hexyl group. Very interestingly, the dark cytotoxicity associated to **COUPY 2** was reduced between 4- and 35-fold in A2780cis and HeLa cells, respectively, when the nanoformulation **NC**-**COUPY 2** was administered. This might be explained by the energy-dependent internalization pathway followed by **NC-COUPY 2** in contrast to **COUPY 2**, which may achieve intracellular accumulation *via* passive diffusion (Figures 5 and S20). Upon red light irradiation, both **COUPY 2** and **NC-COUPY 2** achieved high photoactivation (IC_50_^LIGHT^ = 0.18–0.78 μM) in cancer cells, with phototoxic indexes (PI) up to 255.1 for **NC-COUPY 2** in HeLa cells (Table 1 and Figure 6). Overall, these results indicated that nanoencapsulation of the coumarin PS resulted in decreased dark cytotoxicity and improved in vitro photoactivity with biologically compatible and highly penetrating red light. In addition, it is noteworthy that **NC-COUPY 2** also showed lower cytotoxicity than free coumarin **2** in renal BGM cell line under the dark, which suggest that encapsulation could reduce undesired toxicity on normal dividing cells.

**Table 1:** Phototoxicity of the Compounds toward Cancer and Normal Cells upon Red Light Irradiation Expressed as Mean IC_50_ Values (μM) of Three Independent Measurements^a^

	HeLa			A2780cis			BGM
	dark	light	PI^b^	dark	light	PI^b^	dark
COUPY 1	>200	16 ± 2	>12.5	>200	10.7 ± 0.9	>18.7	>200
COUPY 2	5.7 ± 0.4	0.18 ± 0.01	31.7	5.9 ± 0.9	0.75 ± 0.02	7.9	2.2 ± 0.1
NC-COUPY 2	199 ± 14	0.78 ± 0.09	255.1	20 ± 2	0.7 ± 0.1	28.6	6 ± 1

a Cells were treated for 1.5 h (0.5 h of incubation and 1 h of red irradiation at doses of 89 mW/cm^2^), followed by 48 h of incubation in drug-free medium under normoxia (21% O_2_). Dark analogues were directly kept in the dark for 1 h.

b PI (phototoxic index) = IC_50_ (nonirradiated cells; dark)/IC_50_ (irradiated cells; red light).

Considering that the highest photoactivation using red light for **NC-COUPY 2** was obtained in the HeLa cell line (Figure 6), we conducted a series of experiments reducing red light exposure from 1 h to 0.5 h to evaluate the influence of time during treatments on these cells (Table S1). Compared to 1 h irradiation, slightly high IC_50_^LIGHT^ values were obtained for both free and encapsulated **COUPY 2** when 0.5 h of light exposure was applied, suggesting that the photodynamic effects might be time-dependent. Moreover, 1 h dark cytotoxicity in HeLa cells was found to be similar to those previously obtained with 1.5 h, which led us to think that the cytotoxicity exerted by both **COUPY 2** and **NC-COUPY 2** in the dark was produced shortly after administration to monolayer cells in culture.

**Figure 7:**
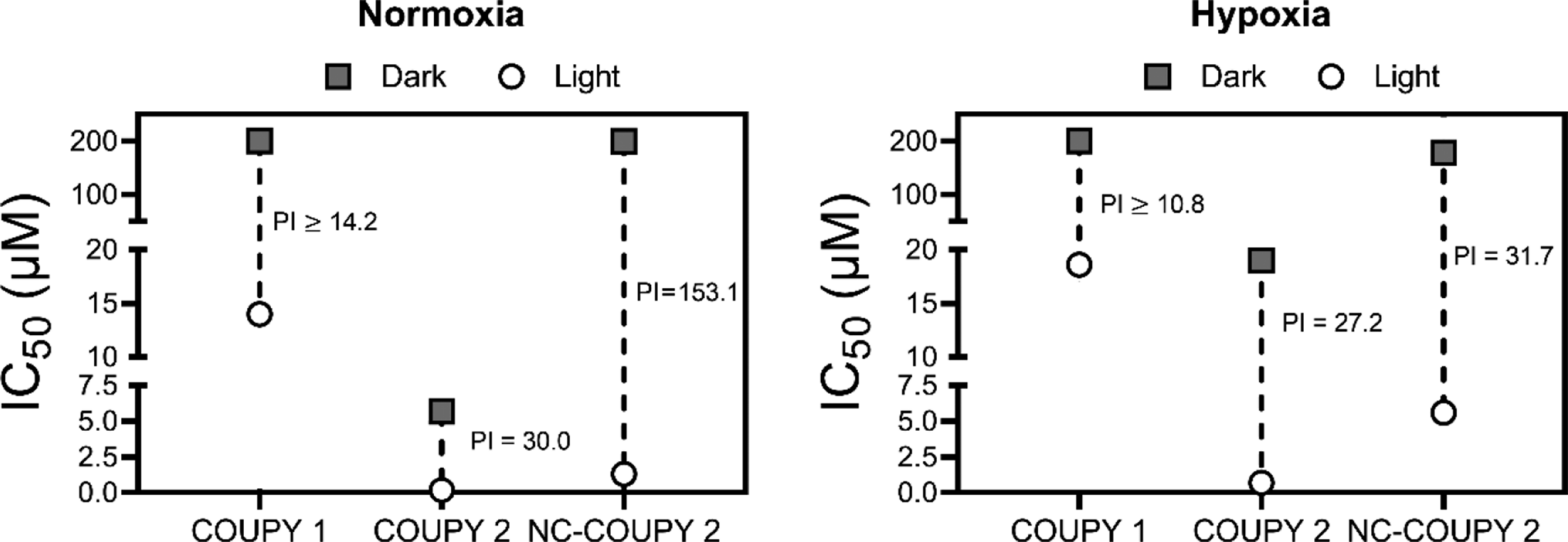
Comparison of half-maximal inhibitory concentration (IC_50_) and PI values for light-activated COUPY compounds (0.5 h in dark + 1 h visible light irradiation followed by 48 h drug-free recovery period) under normoxia (21% O_2_) and hypoxia (2% O_2_) in HeLa cells.

Because these compounds absorb light in the visible region of the electromagnetic spectrum, we decided to investigate photoactivation under broadband visible light instead of using monochromatic red light. This also allowed us to compare their phototoxicity with our previously reported family of COUPY PSs because similar protocols were used.^31^ As shown in Figure 7 and Table S2, PI values for both free coumarins (**1** and **2**) and the encapsulated nanoformulation of **2** in HeLa cells under visible light were comparable to those obtained with red light irradiation in normoxia, being much higher for **NC-COUPY 2** (153.1) than for **COUPY 2** (30), which again demonstrated the positive effect of nanoencapsulaton on the phototoxicity of the PS. It is worth noting that red light lamps delivered high intensity (89 mW/cm^2^ at λ_max_ = 630 nm), whereas visible light irradiation was applied at a much lower intensity (close to 3 mW/cm^2^ at λ_max_ = 520 nm). However, similar IC_50_^LIGHT^ values were obtained (0.19–1.3 μM with visible light compared to 0.18–0.78 μM with red light) for free and encapsulated forms of coumarin **2**. From this, it was clear that COUPY PSs can achieve high photoactivation with low doses of visible light in the wavelength range where they absorb.

Compared to normal oxygen conditions, a reduction in the photoactivity of **NC-COUPY 2** was observed under hypoxia after visible light irradiation (Figure 7). This was probably due to impaired PDT reactions in the low-oxygen environment. Nonetheless, IC_50_^LIGHT^ values were still in the low micromolar range (0.7–5.6 μM), suggesting that the coumarin derivative could still exhibit anticancer photoactivity under low oxygen conditions.

#### 3.4.2 Phototoxic Activity Evaluation on 3D Multicellular Tumor Spheroids

**Figure 8:**
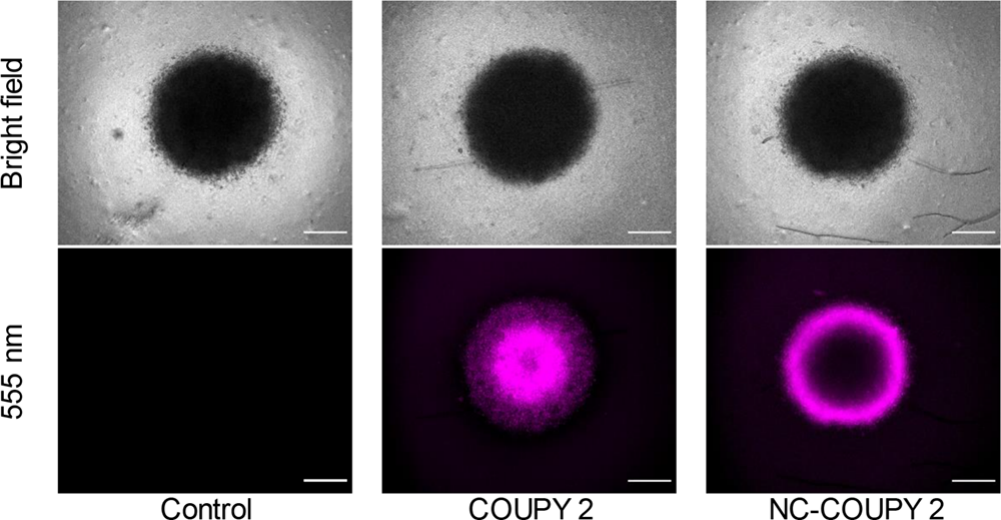
Fluorescence microscopy images of HeLa spheroids treated with **COUPY 2** and **NC-COUPY 2** at 2 μM for 2 h. Scale bar: 100 μm.

After the evaluation of the photocytotoxicity of both **COUPY 2** and **NC-COUPY 2** on 2D monolayer cells, their photoactivity on 3D MCTS was investigated. MCTS represents a closer model to real tumors and can give information about drug penetration into tumoral tissues.^43^ First, the penetration of the compound inside MCTS was examined because COUPY derivatives have demonstrated to act as fluorescent tools that exhibit rapid intracellular accumulation.^31^ Fluorescence microscopy imaging revealed that **NC-COUPY 2** and **COUPY 2** penetrated efficiently into tumor spheres and emitted strong fluorescence (Figures 8 and S18). Interestingly, in contrast to **COUPY 2** fluorescence, which was found evenly distributed across MCTS, **NC-COUPY 2** fluorescence was mostly found on the outer surface of MCTS after 2 h (Figure 8). Nonetheless, increasing the incubation time up to 6 h resulted in complete penetration inside tumor spheres (Figure S21). This delay in complete drug penetration of **NC-COUPY 2** compared to free **COUPY 2** would also imply a reduction in dark cytotoxicity toward MCTS.

**Figure 9:**
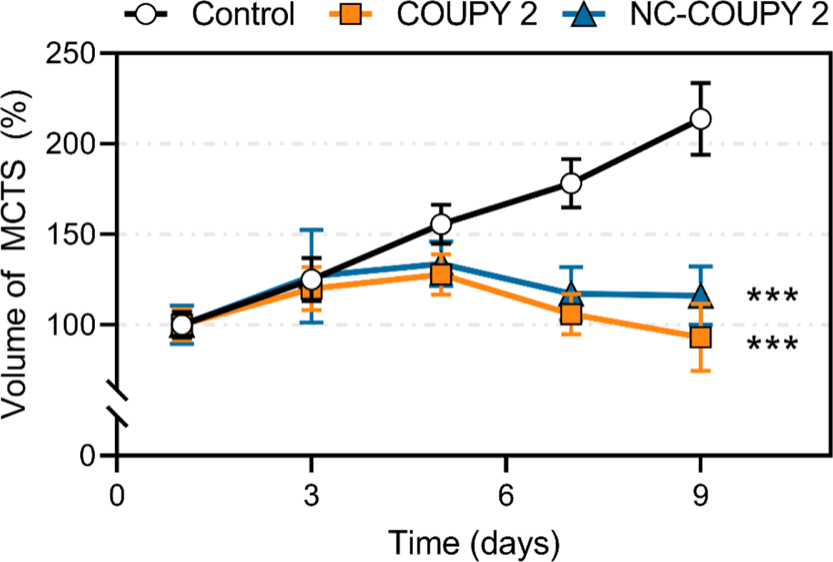
Normalized volume of HeLa MCTS over a span of 9 days. MCTS were treated on day 3 with **COUPY 2** or **NC-COUPY 2** (2 μM) for 6 h in the dark and then exposed to red light irradiation (630 nm, 0.5 h, 89 mW/cm^2^). Data expressed as mean ± SD from three replicates. An independent unpaired *t*-test was used to define statistical differences between the values obtained on day 9 (***p < 0.001).

Following this, the tumor growth of HeLa MCTS was monitored after red light irradiation with **COUPY 2** either free or encapsulated. After the formation of the tumor spheres on day 3, the compounds were incubated for 6 h in the dark as this time was shown to be required for complete penetration into tumor spheres (Figure S21) Then, MCTS were exposed to 0.5 h of red light irradiation at doses of 89 mW/cm^2^. Drug-containing medium was removed, and the volume of the MCTS was monitored over a span of 9 days. Unlike nontreated control cells, the volume of **COUPY 2** and **NC-COUPY 2**-treated MCTS was significantly reduced after light irradiation and provided shrank tumor spheres within the following days until day 9, thereby indicating a potent tumoral growth inhibition effect (Figures 9 and S22). It is noteworthy that similar inhibitory effects on 3D MTCS were found with both free and encapsulated agent after irradiation. These results correlated with those observed in 2D monolayer cells, where similar IC_50_^LIGHT^ were obtained. Overall, this allowed us to confirm the photoactivity of both **COUPY 2** and **NC-COUPY 2** in 3D cellular models, where hypoxia plays a more realistic role than in 2D cell cultures.

#### 3.4.3 Photogeneration of ROS in 2D and 3D Cancer Models

**Figure 10:**
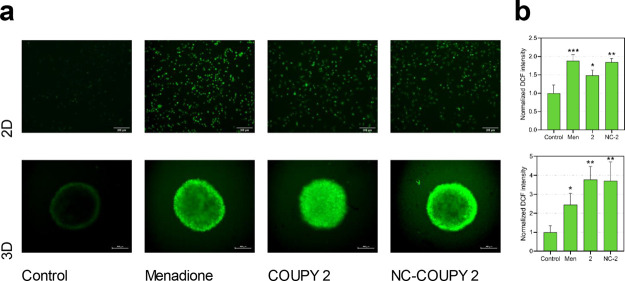
ROS generation in HeLa cells after light irradiation treatments with **COUPY 2** and **NC-COUPY 2** at 2 μM (1 h incubation + 1 h visible light irradiation). (a) ROS levels of HeLa cells on 2D monolayer cells or 3D MCTS stained with DCFH-DA for 0.5 h at 310 K after phototreatments and imaged on a Zeiss Axiovert inverted microscope; menadione (50 μM) being used as positive control. Scale bar: 200 μm. (b) Quantitation of oxidative stress based on DCF fluorescence after irradiation treatments. Three independent experiments were performed, and the error bars were calculated as the SD from the mean. Statistical significance control vs treatments determined *via* one-way ANOVA test (*p < 0.05; **p < 0.01 and ***p < 0.001).

To visualize intracellular ROS generation from the coumarin-based PS, either free or nanoencapsulated, HeLa cells treated with **COUPY 2** or **NC-COUPY 2** at 2 μM upon light irradiation were stained with a 2′,7′-dichlorofluorescein diacetate (DCFH-DA) probe. DCFH-DA is enzymatically converted to the green, fluorescent product DCF in the presence of ROS. Menadione was used as positive control for ROS generation.^44^ The results depicted in Figure 10a proved that **NC-COUPY 2** effectively raised ROS levels in tumor cells in 2D cultures after visible light irradiation. In contrast, although still significant compared to control cells, a weaker green fluorescence was observed for **COUPY 2**-treated cells, suggesting slightly lower ROS generation efficiency in monolayer cells (Figure 10b).

This ROS generating ability was also investigated on MCTS because, as previously indicated, they simulate clinical conditions of tumors such as hypoxia and metabolic gradients to the center.^43^ Treatments with both free and encapsulated agents managed to significantly raise ROS levels after visible light irradiation compared to untreated MCTS (Figure 10a). Interestingly, DCF fluorescence was observed both in the center and in the outer sphere of **COUPY 2**-phototreated MCTS, whereas images of tumorspheres treated with **NC-COUPY 2** showed fluorescence mainly on the outer part. This result is in agreement with the fluorescent penetration pattern observed for the compounds after 2 h (Figure 8). Strikingly, the mean DCF fluorescence intensity was found to be similar for both **COUPY 2** and **NC-COUPY 2** according to quantitative measurement analysis (Figure 10b). Whereas **NC-COUPY 2** only increased DCF fluorescence in the external part of MCTS after irradiation, the overall emission intensity was comparable to those treated with **COUPY 2**, where DCF fluorescence was found across all the tumor spheres. These observations led us to hypothesize that although ROS might not be extensively produced in the hypoxic center of MCTS, a potent ROS generation was achieved with **NC-COUPY 2** in the normoxic outer part of tumor spheres. This also correlated with their phototoxic profile, which resulted in higher PI values in normoxia than under hypoxic conditions (Figures 6 and 7).

Flow cytometry assays using a DCFH-DA probe were also performed to quantitatively analyze ROS generation after phototreatments. As presented in Figure S23, both **COUPY 2** and **NC-COUPY 2** induced large populations of HeLa cells with strong DCF signals compared to control cells. These results correlate well with those previously obtained with fluorescence intensity measurements and corroborated ROS production in cancer cells as a main phototherapeutic mechanism.

#### 3.4.4 Mechanism of Cell Death Induction after Light Irradiation

To gain insights into the cell death mechanisms produced after **NC-COUPY 2** photoactivation, a series of cell-experiments were conducted in HeLa cells. For these experiments, 1 h of visible light irradiation at low doses was applied in order to allow proper comparisons with our previous mechanistic studies with COUPY PSs.^31^ The mechanism of action-related experiments with **COUPY 2** and **NC-COUPY 2** were performed at concentrations close to IC_50_^LIGHT^ with visible light (i.e., 0.5 and 1.5 μM, respectively).

##### 3.4.4.1 Mitochondrial Dysfunction

**Figure 11:**
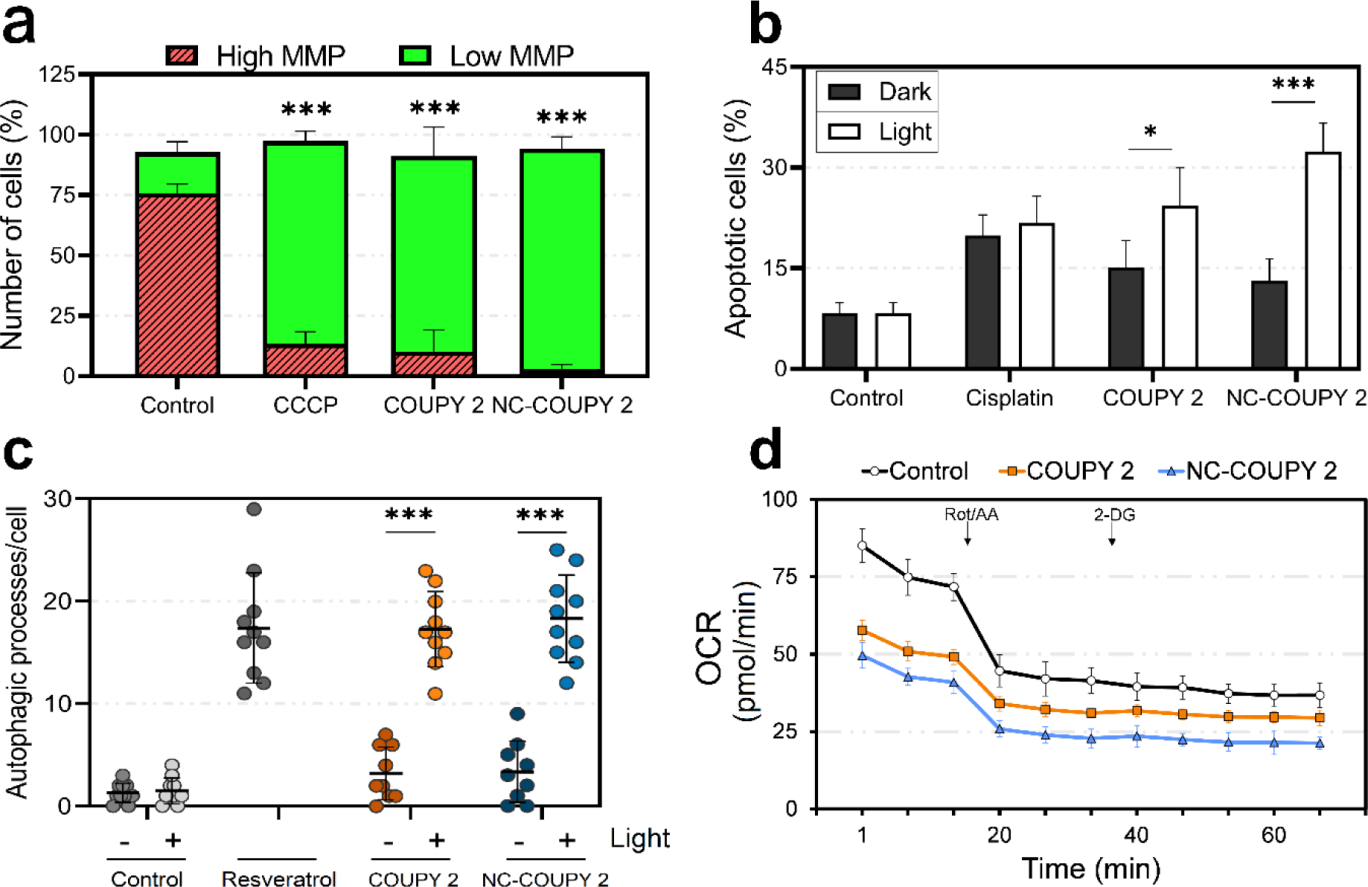
Phototoxic mechanism of action in HeLa cancer cells after treatments with **COUPY 2** or **NC-COUPY 2** at IC_50_^LIGHT^ concentrations (0.5 h incubation + 1 h visible light irradiation and 24 h recovery). (a) Flow cytometry analysis of the MMP using JC-1 dye. The mitochondrial phosphorylation inhibitor carbonyl cyanide *m*-chlorophenyl hydrazone (CCCP 50 μM, 24 h) was used as a positive control for mitochondrial dysfunction. (b) Apoptosis induction upon exposure to **COUPY 2** or **NC-COUPY 2** in the dark or after visible light irradiation treatments detected by flow cytometry as Annexin V-FITC fluorescence on the FL1-H channel; cisplatin (20 μM) was used as the positive control. (c) Number of autophagic processes detected in HeLa cells as quantified by confocal microscopy imaging through monodansylcadaverine (MDC) staining from >10 cells; resveratrol (50 μM, 2 h) was used as the positive control. (d) Mitochondrial oxidative phosphorylation on the basis of the OCR after 2 h treatment with tested complexes (10 μM) in the dark using the Seahorse XFe analyzer. All data represented as mean ± SD from three independent experiments. Statistical significance was determined via two-way ANOVA tests (*p < 0.05; ***p < 0.001).

As shown in Figure 5, mitochondria were found to be the targeted organelle for these family of COUPY derivatives.^31^ Therefore, mitochondrial dysfunction was examined after light irradiation. JC-1 dye was used to assess MMP and mitochondrial health of HeLa cells upon treatments. This dye accumulates in healthy mitochondria in a potential-dependent fashion emitting red fluorescence but exhibits green fluorescence if membrane depolarization occurs. As shown in Figures 11a and S24, both **COUPY 2** and **NC-COUPY 2** dramatically decreased red to green fluorescence ratio after light irradiation, indicating a loss of MMP.

##### 3.4.4.2 Apoptosis Induction

Our previous studies with COUPY derivatives showed that they could act as apoptotic inducers in HeLa cells after visible light irradiation.^31^ To check apoptosis-mediated cell death photoactivation by **NC-COUPY 2**, flow cytometry experiments were performed using Annexin V-FITC (fluorescein isothiocyanate) staining. As shown in Figure 11b, **COUPY 2** and **NC-COUPY 2** produced low to moderate apoptosis levels in the dark, while significant apoptosis induction occurred after irradiation. Interestingly, cell populations with high Annexin V-binding capacity were raised to a larger extent when the nanoformulated agent was applied, suggesting that encapsulation contributed to trigger apoptosis in higher levels (Figures 11b and S25). Along with the depletion of MMP, these findings pointed out an apoptosis induction *via* the mitochondrial intrinsic pathway produced by **NC-COUPY 2.**

##### 3.4.4.3 Autophagy Initiation

To understand cell death mechanisms mediated by **NC-COUPY 2** against HeLa cells after light application, autophagy initiation was investigated. The detection of autophagic processes was performed with monodansylcadaverine(MDC), a probe that accumulates in the acidic compartments of autophagic vesicles; and resveratrol served as a chemical autophagy inducer.^37^ Confocal microscopy imaging revealed that the number of MDC-labeled vesicles significantly increased upon irradiation with both **COUPY 2** and **NC-COUPY 2** (Figures 11c and S26). This is in good correlation with our previously reported results, where pretreatment with the autophagy inhibitor wortmannin was found to significantly attenuate **COUPY 2** phototoxicity.^31^

##### 3.4.4.4 Cell Metabolic Alteration

Because cancer cells generally exhibit a distinct metabolism characterized by producing ATP from glycolysis rather than from mitochondrial oxidative phosphorylation (OXPHOS),^45^ these two major metabolic pathways were studied to assess the bioenergetic state of HeLa cells in real-time using the Seahorse XF-96 flux analyzer. The OCR was used to monitor mitochondrial energetics, whereas glycolysis was evaluated by means of extracellular acidification rate (ECAR) measurements. Treatment for 2 h with both **COUPY 2** and **NC-COUPY 2** resulted in the impairment of mitochondrial respiration as evidenced by reduced OCR before and after the injection of respiratory chain inhibitors (Figure 11d). This is in agreement with MMP depolarization observed upon treatments with these agents.^31^ In addition, ECAR measurements revealed a strong decline in glycolytic function in the presence of these agents, thus revealing strong abrogation of normal cell metabolism (Figure S27).

##### 3.4.4.5 Cell Cycle Distribution

Additionally, the progression of the cell cycle of HeLa cancer cells was examined using propidium iodide staining after irradiation treatments with **NC-COUPY 2** (Figure S28). Compared to cisplatin, which produced S and G2/M phase arrest, **COUPY 2** and **NC-COUPY 2** did not alter cell cycle distribution significantly in the dark. However, light exposure triggered significant accumulation of HeLa cells in the subG1 phase, an indicative of fragmented DNA probably derived from apoptotic cell death induction.

Because both autophagy and mitochondrial dysfunction were observed after irradiation with these compounds (Figure 11), we hypothesize that mitophagy might occurr as a result of cellular photodamage. In fact, this was observed under confocal microscopy upon laser beam irradiation (Figure S19)^31^ and is consistent with the depleted MMP and declined OCR observed after treatment with **COUPY 2** and **NC-COUPY 2** (Figure 11). The mitochondrial photodamage induced by this PS agent could then trigger both apoptotic cell death and mitochondrial degradation through autophagy. Altogether, these results showed that the mechanism of the action of **COUPY 2** involved a combination of autophagy and apoptosis, which may arise from ROS-generating PDT reactions. This mode of cell death was induced in a greater extent when nanoformulation **NC-COUPY 2** was applied, suggesting that the encapsulation of **COUPY 2** improved the phototherapeutic activity of the PS, probably due to the increased amount of the PS being delivered into cancer cells at a time *via* active transport.

## 4 Conclusions

In summary, we have demonstrated that polyurethane–polyurea hybrid NCs can be used to efficiently encapsulate low-molecular-weight PSs based on organic fluorophores for application as nanoPDT agents. As a proof-of-concept, two mitochondria-targeted PS agents based on *N*-alkylpyridinium COUPY coumarins (**1** and **2**) were selected to set up the nanoencapsulation process. Although both coumarins could be encapsulated, the *N*-methyl analogue (**1**) was lost from the NC during the dialysis purification, which indicates that higher hydrophobicity is required to generate stable COUPY-loaded NCs. By contrast, the *N*-hexyl-containing COUPY **2**-loaded NCs (**NC-COUPY 2**) showed a high cargo loading content, as determined by UV–vis spectroscopy, and a controlled particle size distribution of approximately 14 nm with a roughly round shape according to DLS analysis and TEM micrographs, respectively. To our delight, the hydrophobic environment provided by the NCs around the cargo had a positive effect in some key photophysical properties for bioimaging applications. On the one hand, **COUPY 2**-loaded NCs showed a deep pink color owing to an intense absorption band centered around 555 nm, which was slightly redshifted with respect that of the free coumarin in H_2_O. Similarly, the fluorescence quantum yield of **NC-COUPY 2** was higher than that of the nonencapsulated compound in H_2_O. On the other hand, encapsulation had a clear positive effect on the photostability of the coumarin PS in PBS under green light irradiation. Singlet oxygen generation was slightly more efficient when the coumarin was encapsulated, thereby suggesting that nanoencapsulation in a hydrophobic environment has also a positive effect on type II PDT photochemical reactions, leading to the generation of singlet oxygen.

Confocal microscopy revealed that an enabled active transport was involved in the cellular internalization of the NCs and that the released **COUPY 2** accumulates preferentially in the mitochondria. Our in vitro evaluation analyses showed that nanoencapsulation of the coumarin PS decreased dark cytotoxicity and improved photoactivity with biologically compatible and highly penetrating red light, leading to higher PI values compared with the free compound (255 for **NC-COUPY 2** vs 30 for **COUPY 2**) in normoxia and micromolar efficacy under hypoxia. This reduction in dark cytotoxicity was also observed in normal dividing BGM cells. Importantly, a potent tumor growth inhibition effect against clinically relevant multicellular 3D tumorspheres was found upon red light irradiation. The high phototoxic profile of **NC-COUPY 2** can be explained by strong ROS photogeneration in both 2D and 3D cancer models. Along with mitochondrial photodamage, these ROS-generating PDT reactions triggered apoptotic cell death and mitochondrial degradation through autophagy. The fact that this mode of cell death was induced in a greater extent when nanoformulation **NC-COUPY 2** was applied compared with the free coumarin confirms the potential of polyurethane-polyurea hybrid NCs in the development of novel nanoPDT agents. Work is in progress in our laboratory to explore the encapsulation of NIR PSs to explore clinical applications.

## Supporting Information Available

General methods and analytical techniques. Detailed procedures for the synthesis and characterization (IR, DLS, TEM, Z-potential, UV–vis and fluorescence spectroscopy, and photostability) of coumarin-loaded NCs. Additional figures and tables for their biological evaluation (confocal microscopy, antiproliferative activity in 2D monolayer cells and in 3D multicellular spheroids, mitochondrial potential assessment, apoptosis induction, autophagy detection, cell metabolism measurements, and cell cycle distribution) (PDF)
