# Supplementary material for: Improving Photodynamic Therapy Anticancer Activity of a Mitochondria-Targeted Coumarin Photosensitizer Using a Polyurethane–Polyurea Hybrid Nanocarrier
Source: Biomacromolecules. 2022 Jun 13;23(7):2900–13. doi: 10.1021/acs.biomac.2c00361 (PMC9277592; doi:10.1021/acs.biomac.2c00361)
Supplement: Supplementary file 1 — bm2c00361_si_001.pdf [file bm2c00361_si_001.pdf]

# SUPPORTING INFORMATION

## Improving PDT anticancer activity of a mitochondria-targeted coumarin photosensitizer using a polyurethane-polyurea hybrid nanocarrier

Joaquín Bonelli,[1,2,#] Enrique Ortega-Forte,[3,#] Anna Rovira,[1] Manel Bosch,[4] Oriol Torres,[2] Cristina Cuscó,[2] Josep Rocas,[2] José Ruiz,[3,*] Vicente Marchán[1,*]

[1] Departament de Química Inorgànica i Orgànica, Secció de Química Orgànica, IBUB, Universitat de Barcelona (UB), E-08028 Barcelona, Spain. Email: vmarchan@ub.edu

[2] Nanobiotechnological Polymers Division, Ecopol Tech, S.L., El Foix Business Park, Indústria 7, 43720 L'Arboç del Penedès, Tarragona, Spain

[3] Departamento de Química Inorgánica, Universidad de Murcia, and Institute for Bio-Health Research of Murcia (IMIB-Arrixaca), E-30071 Murcia, Spain. Email: jruiz@um.es

[4] Unitat de Microscòpia Òptica Avançada, Centres Científics i Tecnològics (CCiTUB), Universitat de Barcelona (UB), E-08028 Barcelona (Spain)

# These authors contributed equally

# Table of contents


1. Materials
    1.1. Building blocks and crosslinkers S3
    1.2. Encapsulated coumarins S3
    1.3. Solvents and auxiliary solutions S3
    1.4. Biological agents, mediums and supplements S3

2.- Analytical techniques
    2.1. Infrared spectroscopy (IR) S4
    2.2. pH measurements S4
    2.3. Dynamic light scattering (DLS) S4
    2.4. Transmission electron microscopy (TEM) S4
    2.5. High resolution transmission electron microscopy (HR-TEM) S4
    2.6. Zeta potential (Z-pot) S5
    2.7. Dialysis purification S5
    2.8. Determination of cargo loading by UV-vis spectroscopy S5
    2.9. Solids concentration S5

3.- Synthetic procedures.
    3.1 Synthesis of redox-responsive amphiphilic cationic prepolymer (**P1**) S6
    3.2. Synthesis of COUPY **1**-loaded amphoteric redox responsive NCs (**NC-COUPY-1** S6
    3.3. Synthesis of COUPY **2**-loaded amphoteric and redox responsive NCs (**NC-COUPY-2**) S7
    3.4. Synthesis of non-loaded amphoteric and redox responsive NCs (**NC-GTCC**) S8

4.- Characterization of polymer **P1** and NCs
    4.1. Infrared Spectroscopy S9
    4.2. COUPY loading S11
    4.3. Average size of NCs by DLS S12
    4.4. Transmission electron microscopy (TEM) S13
    4.5.High resolution transmission electron microscopy (HR-TEM) S14
    4.6. Stability of COUPY 2-loaded NCs under reducing conditions S14
    4.7. Z-potential of NCs S15

5.- Photophysical characterization S16

6- Singlet oxygen measurements S18

7.- Fluorescence imaging by confocal microscopy S20

8.- Biological studies S23
    8.1. Photocytotoxicity evaluation in 2D monolayer cells S23
    8.2. Photocytotoxicity evaluation in 3D multicellular spheroids S24
    8.3. Reactive oxygen species generation S25
    8.4.. Mitochondrial membrane potential assessment S26
    8.5. Apoptosis induction S26
    8.6. Autophagy detection S27
    8.7. Cell metabolic measurements S28
    8.8. Cell cycle distribution S29

9.- References S30

# 1. Materials

## 1.1. Building blocks and crosslinkers

Isophorone diisocyanate (IPDI) was purchased from Quimidroga (Barcelona, Spain), YMER N-120 was kindly supplied by Perstorp (Malmö, Sweden) and Genamin TAP 100D (1,3-diamino-*N*-octadecylpropane) was provided by Clariant (Barcelona, Spain). Jeffcat DPA (*N*-(3-dimethylaminopropyl)-*N*,*N*´-diisopropanolamine), DEDS (2-hydroxyethyl disulfide), DETA (diethylene triamine) and L-lysine hydrochloride were purchased from Sigma Aldrich (St Louis, USA).

## 1.2. Encapsulated coumarins

COUPY dyes **1** and **2** were synthesized as previously described.[1] Neobee 1053 (caprilyc/capric triglyceride or GTCC) was purchased from Stepan Company (Illinois, United States).

## 1.3. Solvents and auxiliary solutions

Milli-Q water was obtained from a Merck Millipore purification system (Madrid, Spain) and PBS, HCl 37% and NaOH in pellets were purchased from Merck (Madrid, Spain).

## 1.4. Biological agents, mediums, and supplements

L-Glutathione (GSH) and its oxidized derivative (GSSG) were acquired from TCI Chemicals (Tokyo, Japan). Dulbecco's Modification of Eagle's Medium (DMEM) was purchased from Corning (Arizona, USA) and DMEM with low glucose, pyruvate and without glutamine and Phenol Red were purchased from Thermo Fischer Scientific (Massachusetts, USA), as well as Fetal Bovine Serum (FBS), Horse serum, Hoescht 33342 solution, and Mytotracker Green (MTG). L-glutamine solution (200 mM), F12 (HAM) media and 100 U $mL^{-1}$ penicillin/100 μg $mL^{-1}$ streptomycin were acquired from Biological Industries (Cromwell, USA). Hepes buffer (1M) was purchased from Lonza (Basel, Switzerland). Insulin, Hydrocortisone and Epidermal Growth Factor (EGF) were acquired from Sigma-Aldrich (St Louis, USA). Choleric toxin was purchased from Calbiochem (San Diego, CA, USA).

## 2. Analytical techniques

### 2.1. Infrared spectroscopy (IR)

IR spectra were performed in a Smart ATR (Nicolet iS10, Thermo Scientific, Raleigh, USA) using a transmittance mode (16 scans) and OMNIC software. For the monitoring of solvent-based samples, one drop was deposited onto the diamond crystal and the solvent was left to dry by evaporation. IR spectra were recorded from a dry film of the sample for the reaction control after emulsification.

### 2.2. pH measurements

The pH of the emulsion was determined right after the crosslinker was added and at different time intervals until the last polyaddition reaction was complete. All the determinations were carried out in a pH-meter HI 2211 pH/ORP-Meter (HANNA Instruments, Eibar, Spain) equipped with a pH electrode Crison 5029 (Crison Instruments, Barcelona, Spain) and a temperature probe.

### 2.3. Dynamic light scattering (DLS)

The size distribution of the NCs was analyzed on a Zetasizer Nano-ZS90 (Malvern, Worcestershire, UK) in Milli-Q water at 25 °C at a concentration of 0.5 mg/mL.

### 2.4. Transmission electron microscopy (TEM)

The morphology of nanocapsules was studied on a TEM Jeol J1010 (Peabody, MA, USA) equipped with a CCD Orius camera (Gatan). A 400-mesh copper grid coated with 0.75 % FORMVAR was deposited on 6 µL of a suspension of nanocapsules in water (10 mg $mL^{-1}$) for 25 min. Excess of sample was removed by oblique contact with Whatman filter paper and the grid was deposited on a drop of uranyl acetate (2 % w/w) in water for 30 s. Excess uranyl acetate was removed and the grid was air-dried for at least 3 h prior to measurement.

### 2.5. High resolution transmission electron microscopy (HR-TEM)

HR-TEM observations were performed in a JEOL J2100 microscope (Peabody, MA, USA), operating at an accelerating voltage of 120 kV. Images were recorded using a Gatan Orius CCD camera. A 200-mesh copper grid coated with 0.75% FORMVAR was deposited on 6 µL of a suspension of nanocapsules in water (10 mg $mL^{-1}$) for 1 min. Excess of sample was removed by contact with ultrapure water for 1 min and the grid was deposited on a drop of uranyl acetate (2% w/w) in water for 1 min. Excess uranyl acetate was removed and the grid was air-dried for at least 3 h prior to measurement.

## 2.6. Zeta potential (Z-pot)

The Z-pot of the NCs was analyzed on a Zetasizer Nano-ZS90 (Malvern, Worcestershire, UK) in Milli-Q water at 25 °C at a concentration of 1 mg/mL, measured at different pH values.

## 2.7. Dialysis purification

The NCs were dialyzed against Milli-Q water for 24 h using a Spectra/Por molecular porous membrane tubing with a 12–14 kDa molecular weight cut-off (MWCO) (Spectrum Laboratories, Rancho Dominguez, USA).

## 2.8. Determination of cargo loading by UV-vis spectroscopy

The coumarin loading (or drug-loading, DL) of the NCs was determined by UV-Vis measurements performed in a DINKO UV-6900 spectrophotometer (Dinko Instruments, Barcelona, Spain). First, a calibration curve was developed by preparing a range of standard solutions containing the coumarin at different concentrations and analyzing their UV profile and maximum absorbance. Then, Encapsulation Efficiency EE (%) and Dye Loading DL (%) were calculated from the following equations:

$$\%\,EE = \frac{amount\ of\ COUPY\ compound\ incorporated\ in\ the\ nanocapsule}{total\ amount\ of\ COUPY\ compound\ added\ in\ the\ synthesis} * 100 \quad (1)$$

$$\%\,DL = \frac{amount\ of\ COUPY\ compound\ incorporated\ in\ the\ nanocapsule}{total\ amount\ of\ dried\ nanocapsules} * 100 \quad (2)$$

To determine the amount of coumarin incorporated in the NCs, a desired amount of dried NCs (previously dialyzed) was dissolved in an exact volume of solvent and the measurement was calculated from the calibration curve. Non-loaded NCs were used as the reference. All measurements were recorded at the desired wavelength range, depending on the maximum of absorption of the molecule under study and assayed in triplicates.

## 2.9. Solids concentration

NCs concentration in the aqueous dispersion was determined by triplicate leading to dryness using a Digitheat-TFT oven (J.P.Selecta, Barcelona, Spain), with a fixed temperature of 40 ºC for 48 h.

## 3. Synthetic procedures

### 3.1. Synthesis of redox-responsive amphiphilic cationic prepolymer (P1)

2,2´-Dihydroxyethyl disulfide (901.0 mg, 11.68 meq), YMER N-120 (12.04 g, 23.18 meq) and *N*-(3-dimethylaminopropyl)-*N,N´*-diisopropanolamine (981.3 mg, 8.99 meq) were added into a three-necked round-bottom flask equipped with mechanical stirring at room temperature and purged with $N_2$. When the mixture was homogeneous, isophorone diisocyanate (8.14 g, 73.24 meq) was added into the reaction vessel under gentle mechanical stirring. The polyaddition reaction was kept under these conditions until the NCO stretching band intensity did not change, monitored by IR spectroscopy. At this point, dry THF (21 mL) was added into the reaction mixture to fluidify the polymer. In parallel, 1,3-diamino-*N*-octadecylpropane (5.99 g, 35.45 meq) was dissolved with dry THF (5.23 mL) into another 100 mL three-necked round-bottom flask, which had previously been purged with $N_2$. The former reaction mixture was added dropwise onto the latter under half-moon 100 rpm mechanical stirring. The reaction was monitored by IR until the NCO stretching band intensity had completely disappeared.

### 3.2. Synthesis of COUPY 1-loaded amphoteric redox responsive NCs (NC-COUPY 1)

Isophorone diisocyanate (69.9 mg, 0.63 meq) was added into a three-necked round bottom flask equipped with mechanical stirring, purged with $N_2$ and protected from light. In parallel, COUPY **1** (3.1 mg, 8.06 µmol), Neobee 1053 (14.6 mg, 35.73 µmol), polymer **P1** (655.1 mg, 0.07 meq) and dry THF (0.25 mL) were mixed in a vial, added into the flask and homogenized for 10 min at 150 rpm, protected from light. At this point, an alkaline aqueous solution of L-lysine was prepared by dissolving 0.93 g L-lysine in 11.37 g of Milli-Q water and adjusting pH to 11.0 with alkaline NaOH solutions at 3 M and 1 M (total L-lysine concentration 7.56% by wt). This solution (22.84 mg of L-lysine, 0.27 meq) was added at 250 rpm and the polyaddition reaction was checked after 15 min by IR. Then, the organic phase was emulsified at 300 rpm with cold Milli-Q water (10.11 g) and finally a 10% w/w aqueous solution of diethylenetriamine (9.43 mg, 0.27 meq) was added in order to generate crosslinked NCs from the nano micelles. The stirring was reduced to 100 rpm. This polyaddition reaction was monitored by IR and pH measurements. Once the NCs were formed, THF was removed from the reactor at 35 °C under vacuum and the dialysis purification was carried out using a molecular porous membrane tubing with a 12–14 kDa MWCO. After dialyzing for 24 h, water acquired a pink color, as shown in Figure S1, which indicated that the coumarin was partially released from the NCs.

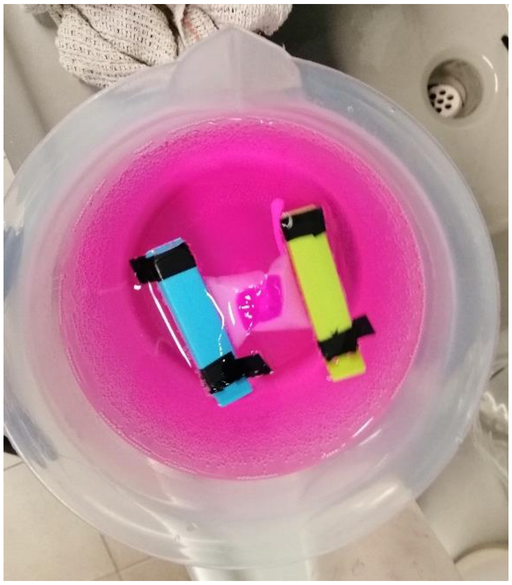

**Figure S1**. Photographic image of the dialysis process of **NC-COUPY 1** after 24 h.

### 3.3. Synthesis of COUPY 2-loaded amphoteric and redox responsive NCs (NC-COUPY 2)

Isophorone diisocyanate (71.1 mg, 0.64 meq) was added into a three-necked round-bottom flask equipped with mechanical stirring, purged with $N_2$ and protected from light. In parallel, COUPY **2** (5.4 mg, 10.89 μmol), Neobee 1053 (24.5 mg, 59.96 μmol), polymer **P1** (877.1 mg, 0.09 meq) and dry THF (0.5 mL) were mixed in a vial, added into the flask and homogenized for 10 min at 150 rpm, protected from light. At this point, an alkaline aqueous solution of L-lysine was prepared by dissolving 0.93 g L-lysine in 11.37 g of Milli-Q water and adjusting pH to 11.0 with alkaline NaOH solutions at 3 M and 1 M (total L-lysine concentration 7.56% by wt). This solution (23.74 mg of L-lysine, 0.28 meq) was added at 250 rpm and the polyaddition reaction was checked after 15 min by IR. Then, the organic phase was emulsified at 300 rpm with cold Milli-Q water (6.70 g) and finally a 10% w/w aqueous solution of diethylenetriamine (7.63 mg, 0.22 meq) was added in order to generate crosslinked NCs from the nano micelles. The stirring was reduced to 100 rpm. This polyaddition reaction was monitored by IR and pH measurements. Once the NCs were formed, THF was removed from the reactor at 35 °C under vacuum and the dialysis purification was carried out for 24 h using a molecular porous membrane tubing with a 12–14 kDa MWCO. As shown in Figure S2, water did not acquire any color, indicating that COUPY **2** was retained inside the NCs during the dialysis purification (compare with Figure S1).

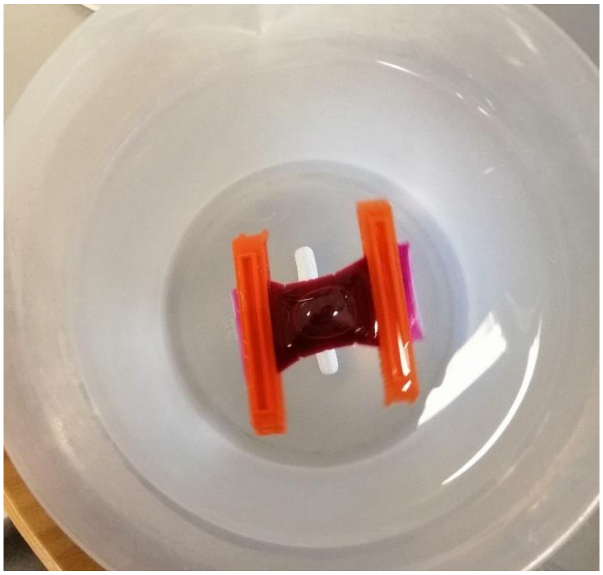

**Figure S2**. Photographic image of the dialysis process of **NC-COUPY2** after 24 h.

### 3.4. Synthesis of non-loaded amphoteric and redox responsive NCs (NC-GTCC)

The procedure was based on the one used for the synthesis of redox responsive COUPY **2**-loaded amphoteric NCs (see section 3.3) with the exception that no coumarin was added to the reaction. The exact amounts of the reagents are detailed in Table S1.

**Table S1**. Amounts of reagents used to prepare **NC-GTCC.**

| Compound | Amount | Equivalents or mols |
|---|---|---|
| **IPDI** | 69.7 mg | 0.63 meq |
| **Neobee 1053 (GTCC)** | 26.3 mg | 56.61 □mol |
| **Polymer (P1)** | 812.5 mg | 0.09 meq |
| **Dry THF** | 1 mL | – |
| **L-lysine** | 20.9 mg | 0.25 meq |
| **Milli-Q water** | 5.93 g | – |
| **DETA** | 7.8 mg | 0.23 meq |

# 4. Characterization of polymer P1 and NCs

## 4.1. Infrared Spectroscopy

The polymerization reaction was easily controlled by IR spectroscopy given that NCO group has a very clear and characteristic stretching band at 2280-2230 cm$^{-1}$.

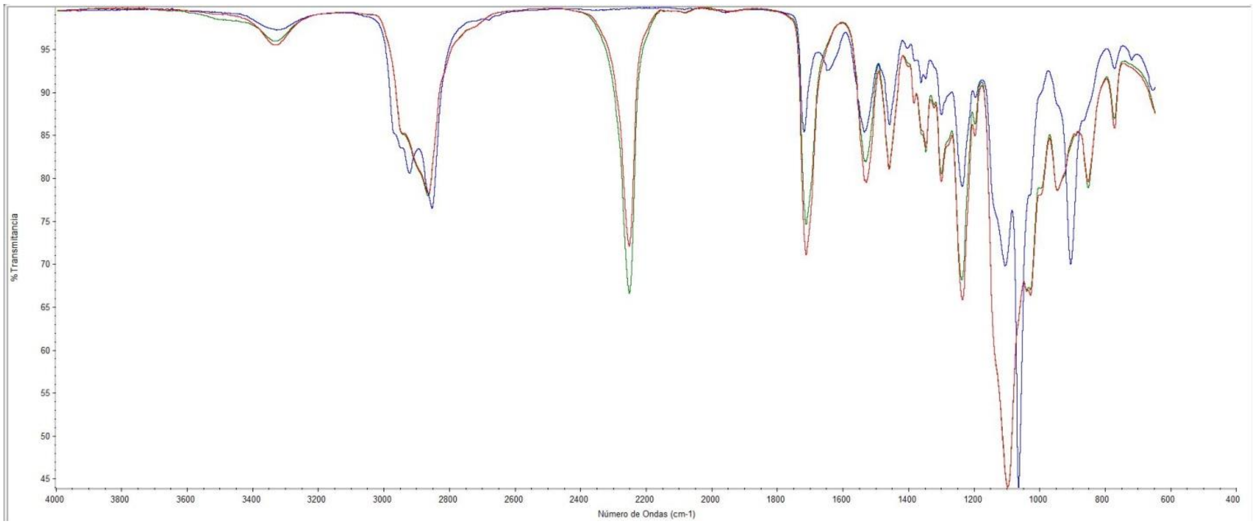


**Figure S3.** Consecutive IR spectra recorded during the synthesis of **P1**.

As shown in Figure S3, IR spectra of the polymer (**P1**) indicated a successful polymerization reaction between diols, the diamine and the diisocyanate, in both steps of the polymer synthesis. The green line corresponds to the first sample recorded, at the begin of the reaction. At that time, the NCO asymmetric stretching band at 2252 cm$^{-1}$ was very sharp and intense. At the end of the first step, involving the reaction between the diols and the diisocyanate (red line), the intensity of the NCO stretching band decreased significantly. Meanwhile, the intensities of the CO stretching band at 1719 cm$^{-1}$, the CN stretching band at 1537 cm$^{-1}$, the NCOO/COC asymmetric stretching band at 1240 cm$^{-1}$ increased. Overall, the IR spectra registered during the first step of the synthesis confirmed polyurethane bond formation along with NCO consumption. Once the diamine was added, during the second step of the polymer synthesis (blue line), the NCO stretching band at 2252 cm$^{-1}$ disappeared instantaneously, which was explained by the high reactivity of the amines. Simultaneously, other characteristic bands appeared or changed, such as a new stretching band at 1634 cm$^{-1}$, which was associated to the carbonyl of urea bonds and a new wagging band at 908 cm$^{-1}$ corresponding to the free secondary amine, which also confirmed polyurea formation.

The encapsulation process was also controlled by IR spectroscopy. As shown in Figure S4, IR spectra of the NCs (regardless their loading) indicated also a successful nanocapsule formation. The blue line in the IR spectra represents the sample 30 min after the polymer, together with the coumarin, was mixed with the diisocyanate. This initial step was the reactivation of the polymer and its conversion to an NCO-reactive entity. Afterwards, L-lysine sodium salt was added (purple line) and reacted with the activated polymer. A decrease on the intensity of the NCO stretching band at 2255 $cm^{-1}$, concomitantly with an increase of the carbonyl and CN stretching bands, confirmed urea formation (1642 $cm^{-1}$ and 1532 $cm^{-1}$, respectively). Finally, the triamine was added (red line) and the NCO stretching band instantaneously disappeared and the urea-associated bands increased their intensity because of the rapid reaction between remaining NCO groups and this polyamine.

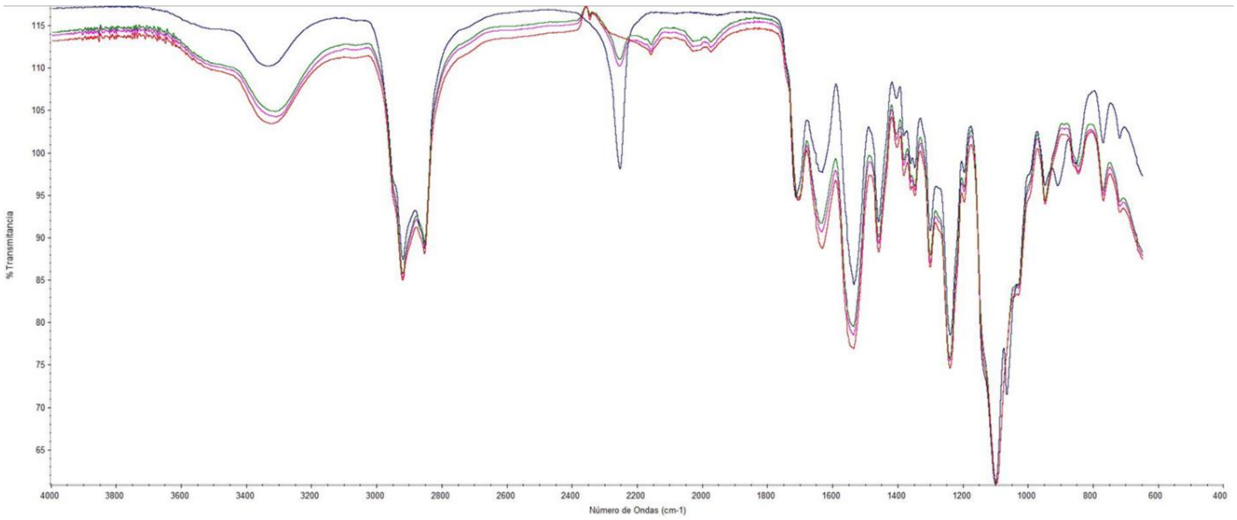


**Figure S4**. Consecutive IR spectra recorded during the encapsulation process with COUPY **2**.

### 4.2. COUPY loading

The concentration of NCs in the final emulsions (mg/mL) was quantified with a solids concentrator, as indicated in section 2.8. Dye Loading (DL) and Encapsulation Efficiency (EE) were determined by UV-Vis spectroscopy following the method described in section 2.7. The calibration plot for COUPY **2** is shown in Figure S8 and the DL and EE parameters for **NC-GTCC**, **NC-COUPY 1** and **NC-COUPY 2** are indicated in Table S3.

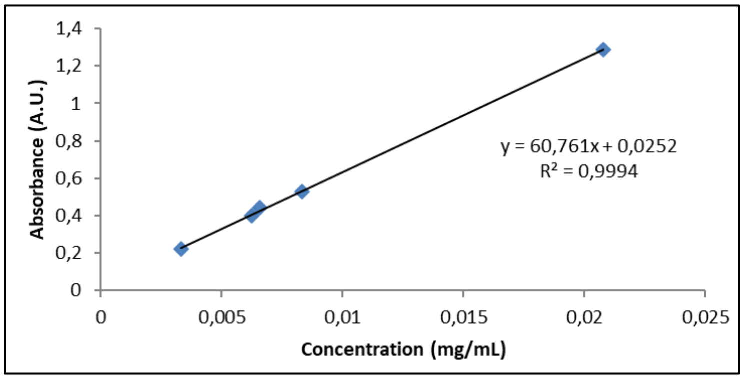


**Figure S5**. Calibration plot for COUPY **2**.

**Table S2**. DL and EE parameters for NCs.

| | **[NCs] (mg/mL)** | **Dye Loading (DL, µM)** | **Encapsulation Efficiency (EE, %)** |
|---|---|---|---|
| **NC-GTCC** | 34.57 ± 1.11 | - | - |
| **NC-COUPY-1** | 41.61 ± 3.63 | Non detected | - |
| **NC-COUPY-2** | 48.45 ± 1.08 | 1157.5 ± 14.0 | 91.2% |

## 4.3. Average size of NCs by DLS

The particle size distribution of the NCs was measured by dynamic light scattering (DLS) (Figures S5 and S6 and Table S2).

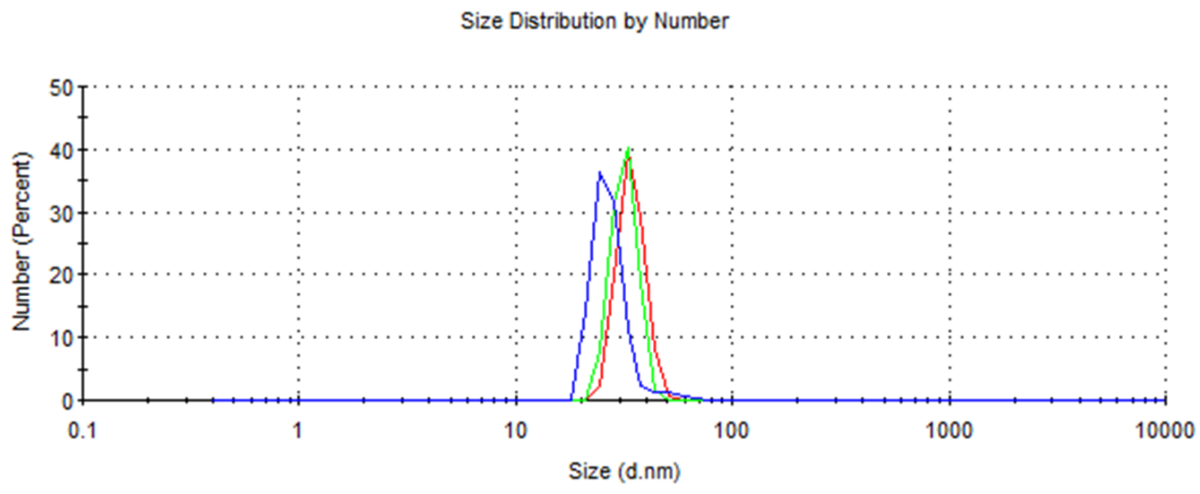


**Figure S6**. Hydrodynamic diameter distribution by number of **NC-COUPY 1**.

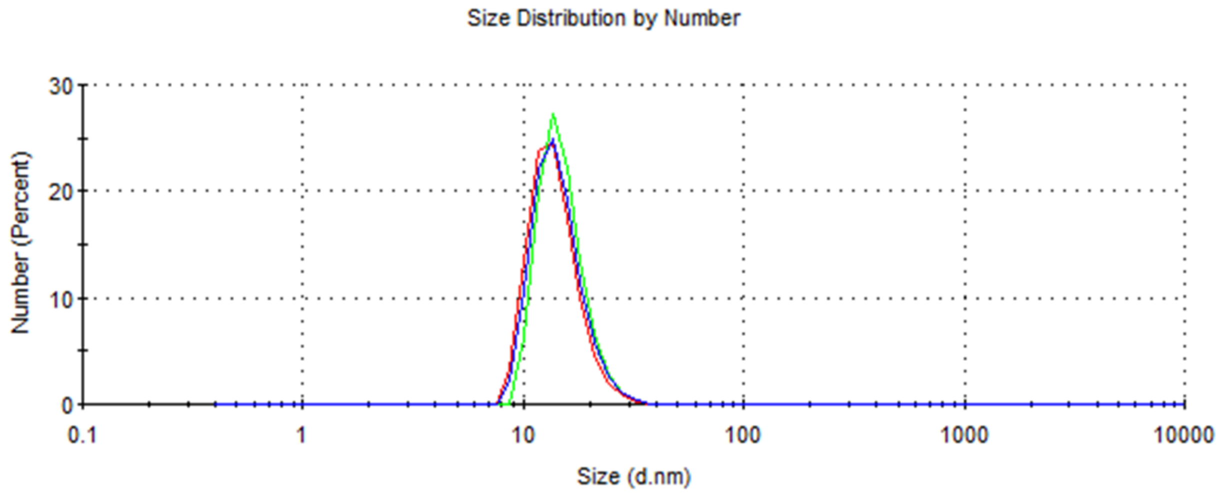


**Figure S7**. Hydrodynamic diameter distribution by number of **NC-COUPY 2**.

**Table S3.** Hydrodynamic diameter average of COUPY-loaded NCs.

| | Experimental Values | Average ± SD |
|---|---|---|
| **NC-COUPY 1** | 34.32 nm | 31.21 ± 3.48 nm |
| | 31.85 nm | |
| | 27.45 nm | |
| **NC-COUPY 2** | 14.04 nm | 14.55 ± 0.53 nm |
| | 15.09 nm | |
| | 14.53 nm | |

## 4.4. Transmission electron microscopy (TEM)

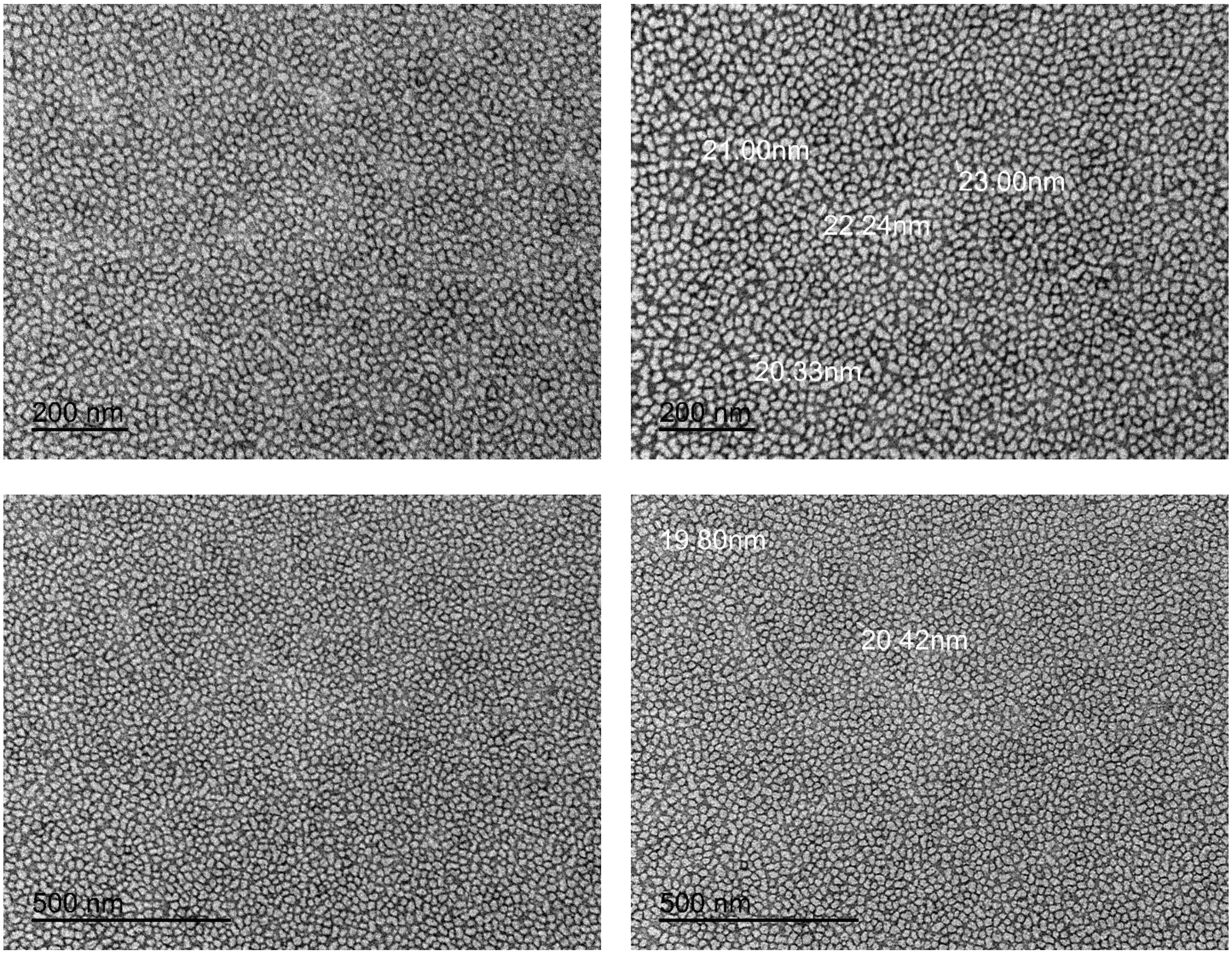


**Figure S8.** Selected TEM micrographs of **NC-COUPY 2** (scale bar: 200 nm and 500 nm).

## 4.5. High resolution transmission electron microscopy (HR-TEM)

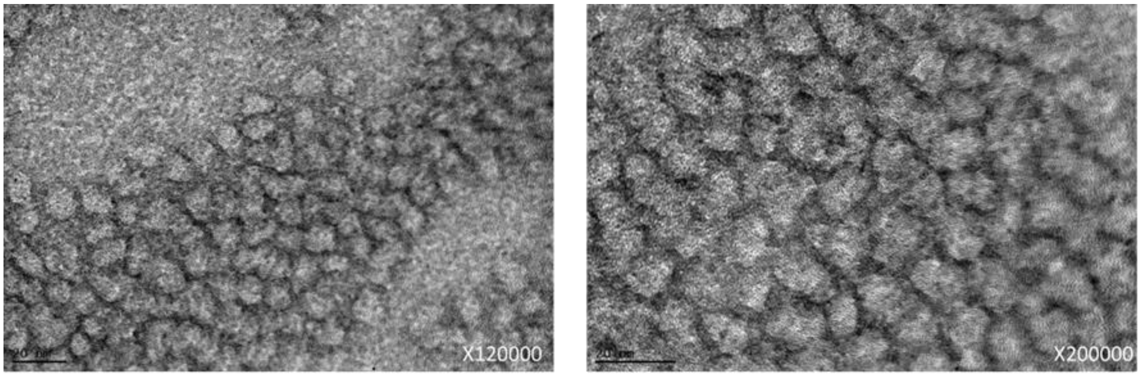


**Figure S9.** Selected HR-TEM images of **NC-COUPY 2** (scale bar: 20 nm) collected at x120000 and x200000 magnifications.

### 4.6. Stability of COUPY 2-loaded NCs under reducing conditions

The degradability of the nanocapsules in PBS supplemented with glutathione (GSH) (10 mM) was studied with the aim of reproducing the situation in the intracellular media of a cancer cell. As shown in Figure S10, incubation of **NC-COUPY 2** in GSH-supplemented PBS for 24 and 48 h at 37 ºC confirmed their degradation and the release of the coumarin PS as inferred with a dialysis methodology. Indeed, the concentration of **COUPY 2** in the dialyzed water, in the case of the PBS-incubated sample, reached a plateau after 24 h of incubation, while the concentration of the coumarin PS in the dialyzed water coming from the GSH-supplemented PBS incubation sample continued increasing over the time.

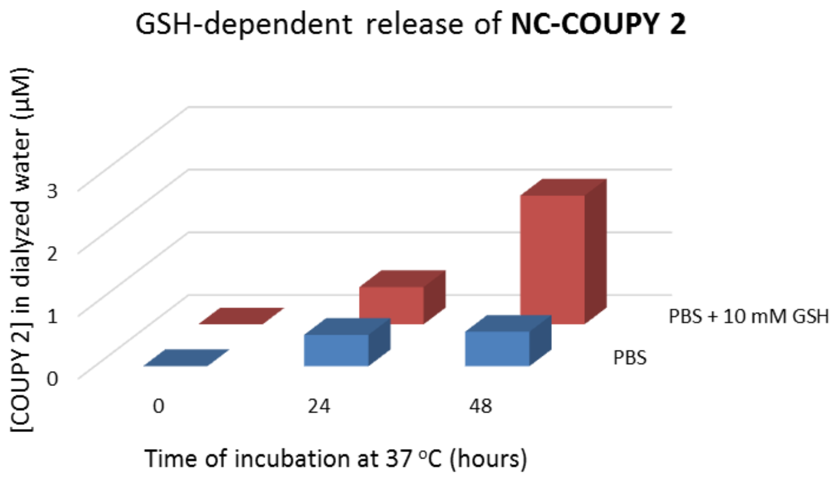


**Figure S10**. Concentration of **COUPY 2** in the dialyzed water of **NC-COUPY 2** in PBS and in PBS supplemented with glutathione at t = 0 and after incubation for 24 h and 48 h at 37 ºC.

## 4.7. Z-potential of NCs

As shown in Figure S9, the Z-potential values (surface charge) of the NCs were determined at different pH values as described in section 2.5.

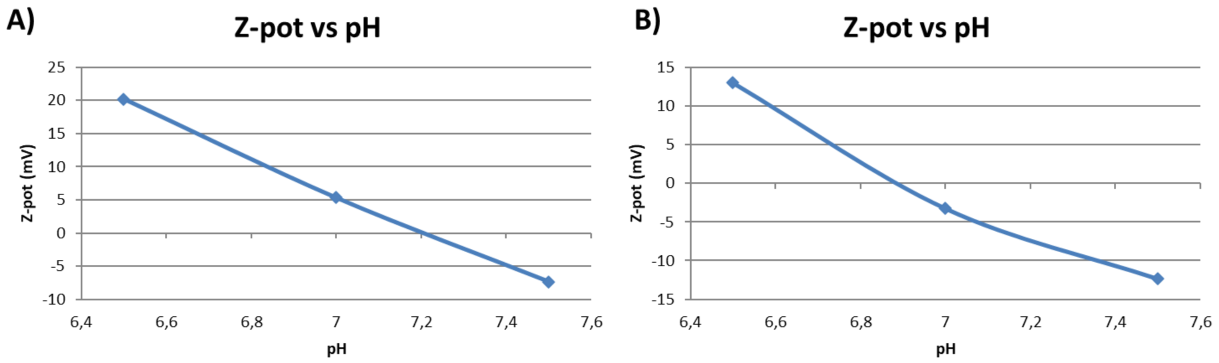


**Figure S11** Z-pot analysis of A) **NCs-COUPY 1** and B) **NCs-COUPY 2** vs pH media

# 5. Photophysical characterization

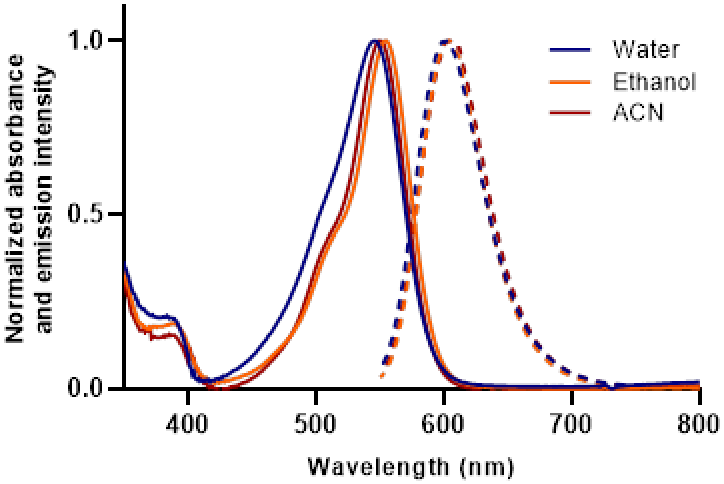


**Figure S12**. Comparison of the normalized absorption (solid lines) and fluorescence (dotted lines) spectra of **COUPY 2** in $H_2O$, ethanol and ACN.

**Table S4**. Photophysical data of **COUPY 2** and of **NC-COUPY-2**.

| Compound | Solvent | $\lambda_{abs}$ [nm][a] | $\lambda_{em}$ [nm][b] | Stokes' shift [nm][c] | $\Phi_F$[d] |
|---|---|---|---|---|---|
| **COUPY 2** | $H_2O$ | 545 | 602 | 57 | 0.20 |
| | Ethanol | 554 | 604 | 50 | 0.47 |
| | ACN | 550 | 604 | 54 | 0.33 |
| **NC-COUPY-2** | $H_2O$ | 550 | 600 | 50 | 0.36 |

[a] Wavelength of the absorption maximum. [b] Wavelength of the emission maximum upon excitation at a wavelength 20 nm below $\lambda_{abs}$. [c] Stokes' shift. [d] Fluorescence quantum yields ($\Phi_F$) were measured by a comparative method using cresyl violet in ethanol ($\Phi_{F;Ref}$ = 0.54) as a reference.

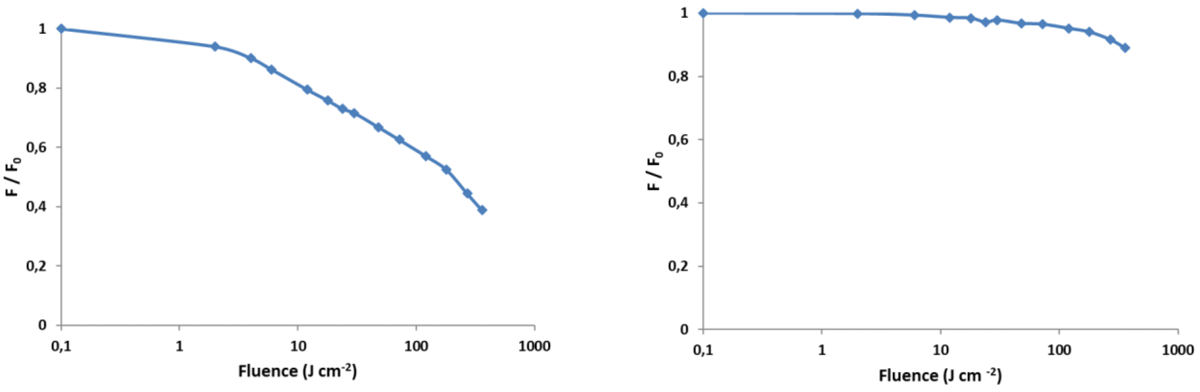


**Figure S13**. Photostability of **COUPY 2** (left) and **NC-COUPY 2** (right) after green LED irradiation at different times expressed as decay of fluorescence emission after subsequent irradiation times.

## 6- Singlet oxygen measurements

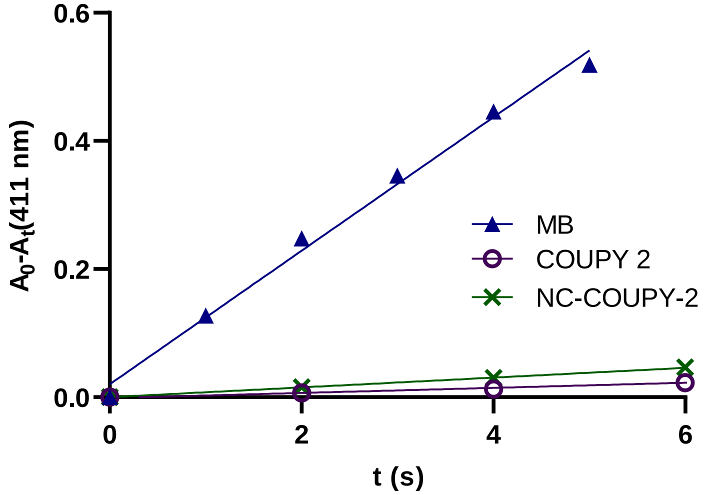


**Figure S14**. Plot of the changes in the absorbance ($A_0$-$A_t$) of DPBF at 411 nm against irradiation time in the presence of the standard photosensitizer methylene blue and the **COUPY 2** and **NC-COUPY 2** in aerated EtOH/$H_2O$ 1:1 (v/v).

A) DPBF

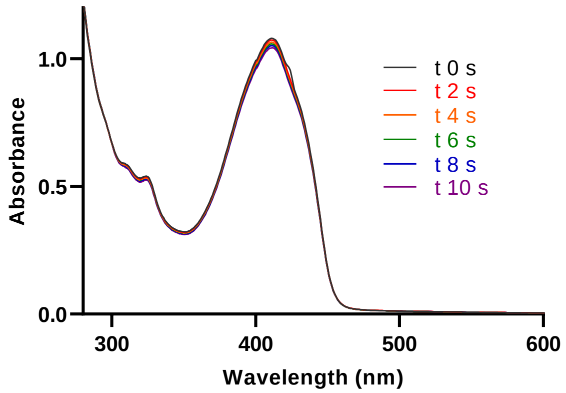

B) MB

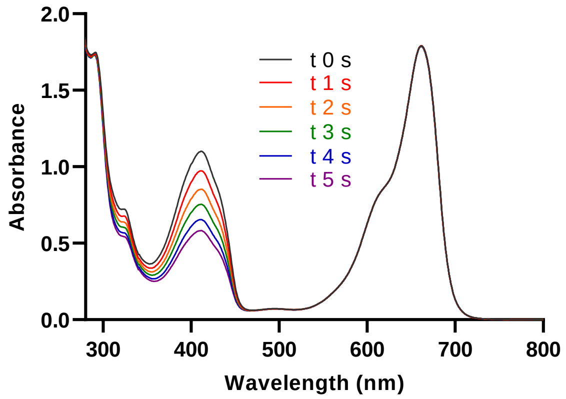


C) COUPY 2

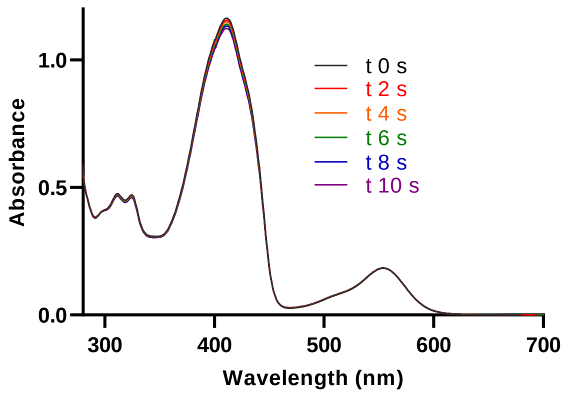


D) NC-COUPY-2

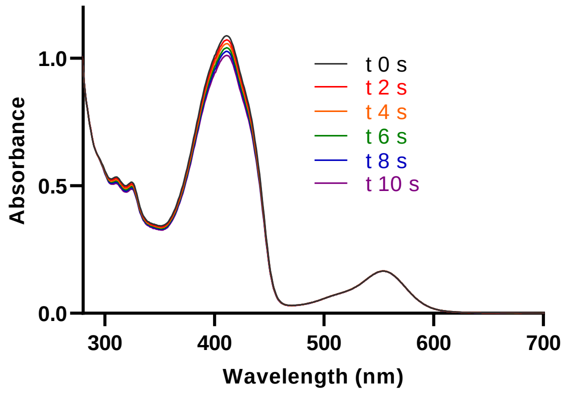


**Figure S15** . Changes in the absorption spectra of DPBF resulting from the irradiation with green LED light in the absence (A) and in the presence of MB (B), **COUPY 2** (C) and **NC-COUPY 2** (D).

## 7. Fluorescence imaging by confocal microscopy

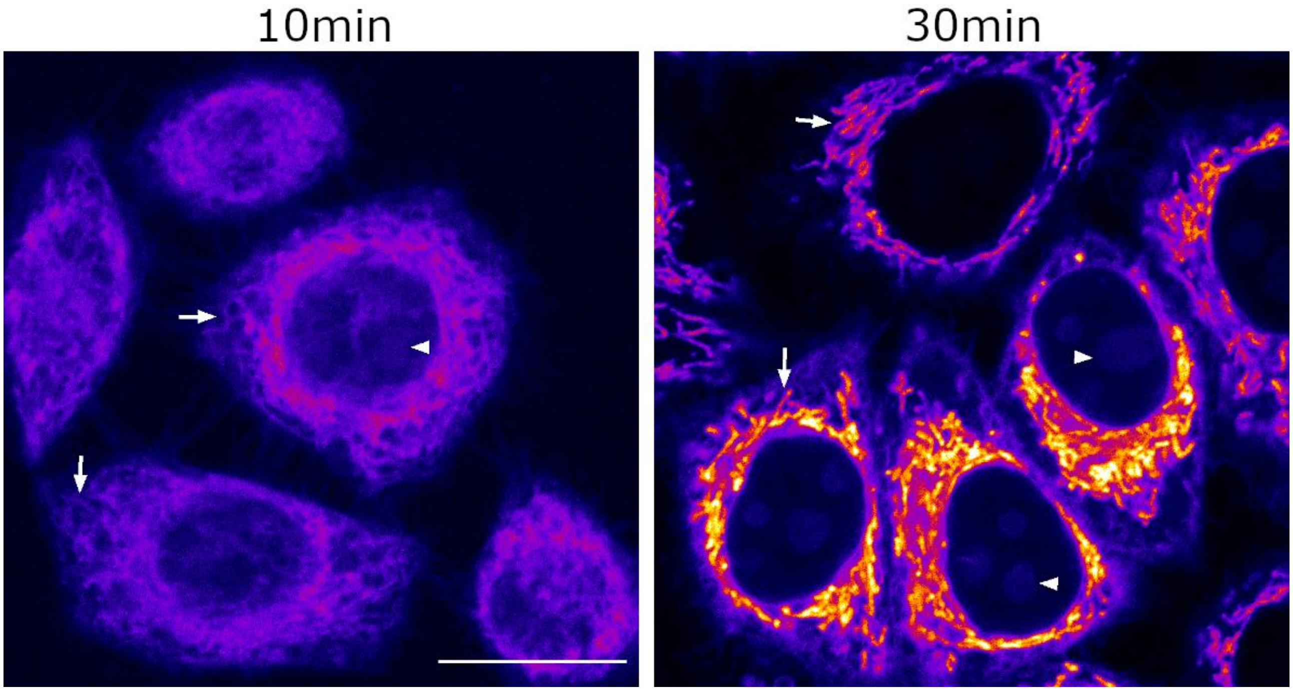


**Figure S16**. Cellular uptake of **NC-COUPY 2**. Single confocal planes of HeLa cells incubated with the NCs (1.0 µM, 37 °C; left. 10 min, right: 30 min). White arrows point out mitochondria and white arrowheads nucleoli staining. Scale bar: 20 µm.

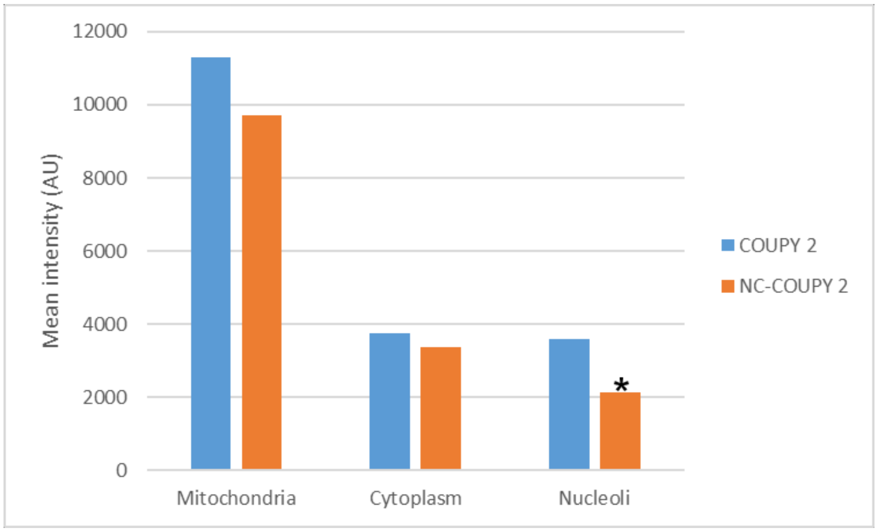


**Figure S17**. Mean intensity (Arbitrary Units) graph of **COUPY 2** and **NC-COUPY 2** at the mitochondria, cytoplasm and nucleoli after incubation with the compounds (1.0 µM, 37 °C, 30 min).

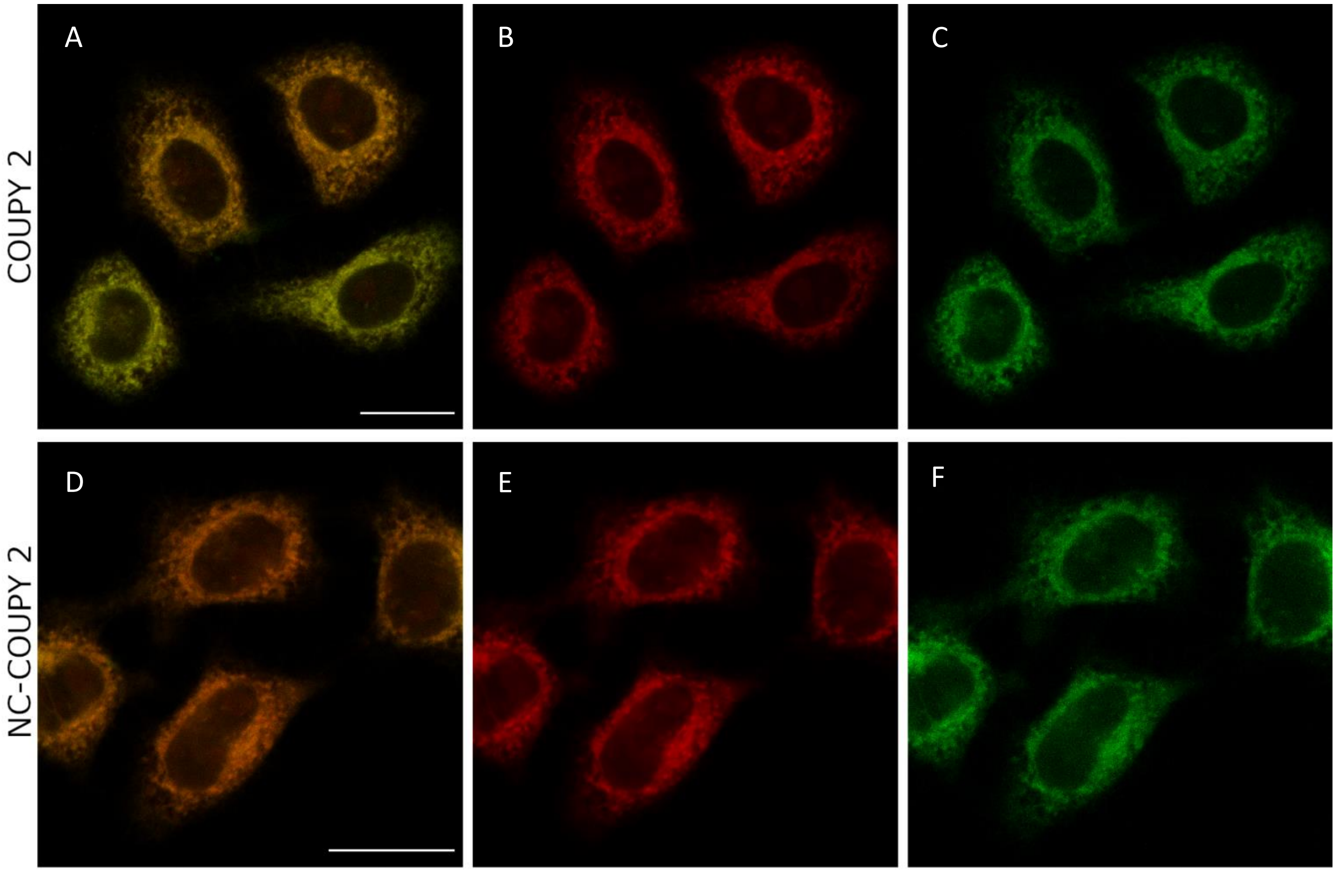


**Figure S18**. Co-localization studies with **COUPY 2** (top) and **NC-COUPY 2** (bottom) and Mitotracker Green FM. Single confocal plane of HeLa cells incubated with **COUPY 2** or **NC-COUPY 2** (1 μM, red) and Mitotracker Green FM (0.1 μM, green). A), D) Overlay of the two staining. B), E) **COUPY 2** and **NC-COUPY 2** signal, respectively. C), F) Mitotracker Green FM signal. Scale bar: 10 μm. All images are at the same scale as A.

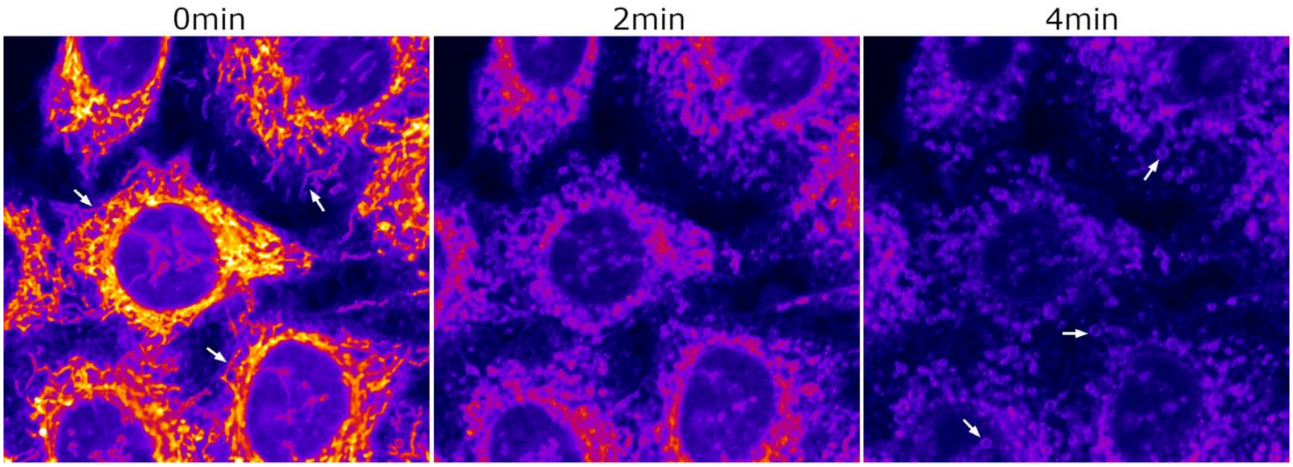


**Figure S19.** Cellular uptake of **NC-COUPY-2**. Single confocal planes at three different time points of image acquisition (left, t = 0; center, t = 2 min; right t = 4 min) are shown after incubation of HeLa cells with the NCs (1.0 μM) during 30 min at 37 °C. White arrows point out mitochondria and donut-shaped mitochondria staining at t = 0 and 4 min, respectively. Scale bar: 20 μm.

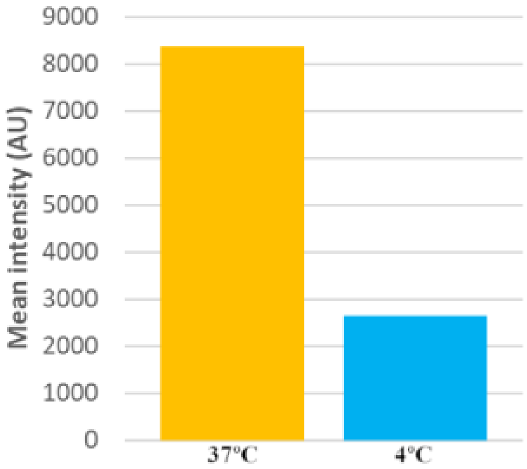


**Figure S20**. Mean intensity (Arbitrary Units) graph of **NC-COUPY 2** after incubation at 37 °C or 4 °C (1.0 μM, 30 min).

# 8. Biological studies

## 8.1. Photocytotoxicity evaluation in 2D monolayer cells

**Table S1**. Phototoxicity of the compounds towards HeLa cancer cells under normoxia upon red light irradiation expressed as mean $IC_{50}$ values (μM) of three independent measurements.[a]

| | Dark | Red light | PI [b] |
|---|---|---|---|
| **COUPY 1** | >200 | 18 ± 2 | >5.5 |
| **COUPY 2** | 5.3 ± 0.3 | 0.76 ± 0.004 | 7.0 |
| **NC-COUPY 2** | 196 ± 15 | 1.8 ± 0.09 | 108.8 |

[a] Cells were treated for 1 h (0.5 h of incubation and 0.5 h of irradiation) followed by 48 h of incubation in drug-free medium. Dark analogues were directly kept in the dark for 1 h.

[b] PI=$IC_{50}$ (non-irradiated cells; dark)/$IC_{50}$ (irradiated cells; red light).

**Table S2**. Phototoxicity of the compounds towards HeLa cancer cells under normoxia (21% $O_2$) or hypoxia (2% $O_2$) upon visible light irradiation expressed as $IC_{50}$ values (μM).[a]

| | | Dark | Light | PI [b] |
|---|---|---|---|---|
| **COUPY 1** | Normoxia | >200 | 14 ± 2 | >14.2 |
| | Hypoxia | >200 | 18.6 ± 0.9 | >10.8 |
| **COUPY 2** | Normoxia | 5.7 ± 0.4 | 0.19 ± 0.03 | 30.0 |
| | Hypoxia | 19 ± 3 | 0.7 ± 0.1 | 27.2 |
| **NC-COUPY 2** | Normoxia | 199 ± 14 | 1.3 ± 0.4 | 153.1 |
| | Hypoxia | 178 ± 23 | 5.6 ± 0.9 | 31.7 |

[a] Cells were treated for 1.5 h (0.5 h of incubation and 1 h of visible irradiation with withe lamps) followed by 48 h of incubation in drug-free medium under normoxic or hypoxic conditions. Dark analogues were directly kept in the dark for 1.5 h.

[b] PI=$IC_{50}$ (dark-non-irradiated cells)/$IC_{50}$ (irradiated cells; white light).

## 8.2. Photocytotoxicity evaluation on 3D multicellular spheroids.

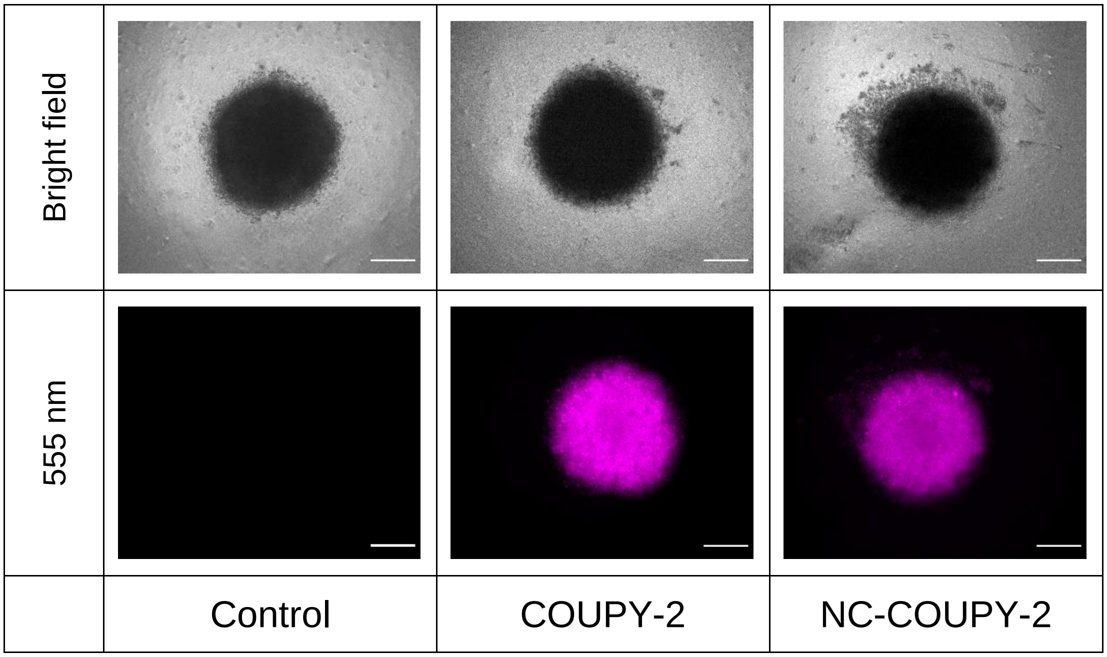


**Figure S21**. Fluorescence microscopy images of HeLa spheroids treated with **COUPY 2** and **NC-COUPY 2** at 2 μM for 6 h. Scale bar: 100 μm.

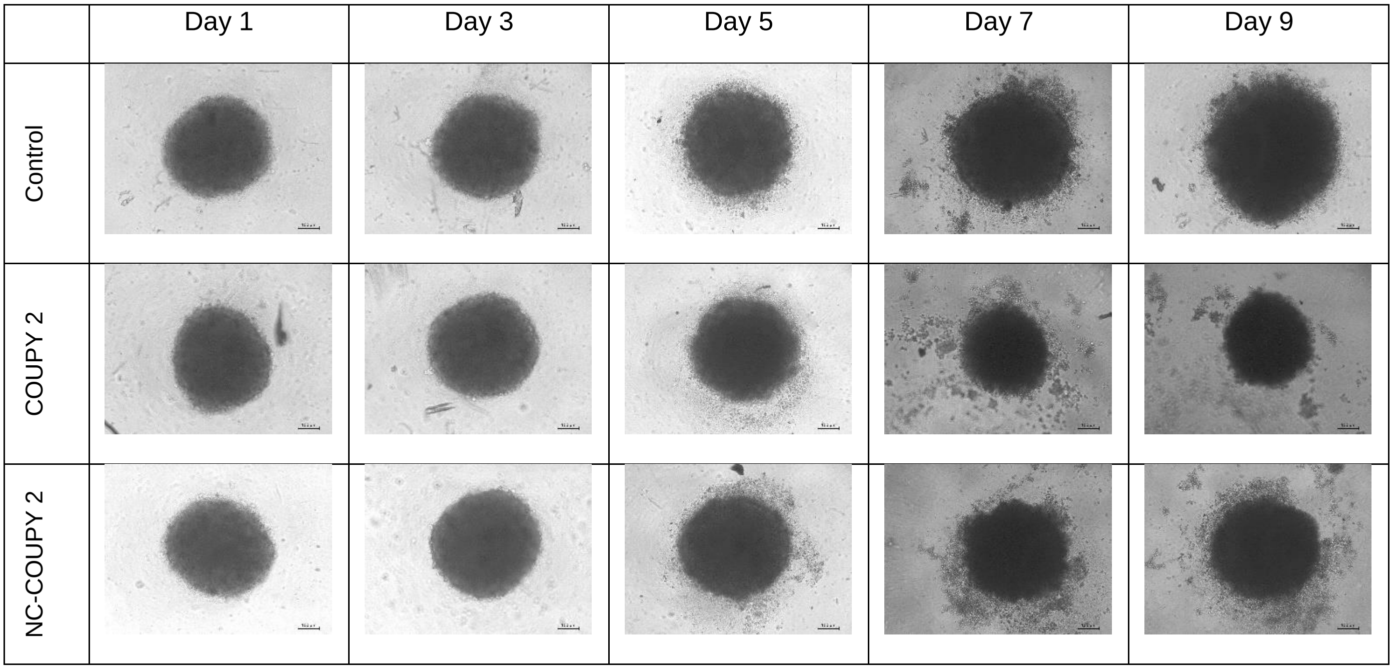


**Figure S22.** Representative microscopy images of HeLa spheroids after treatment with **COUPY 2** and **NC-COUPY 2** at 2 μM (6 h incubation + 0.5 h red light irradiation) on day 3. Scale bar: 100 μm.

### 8.3. Reactive oxygen species generation

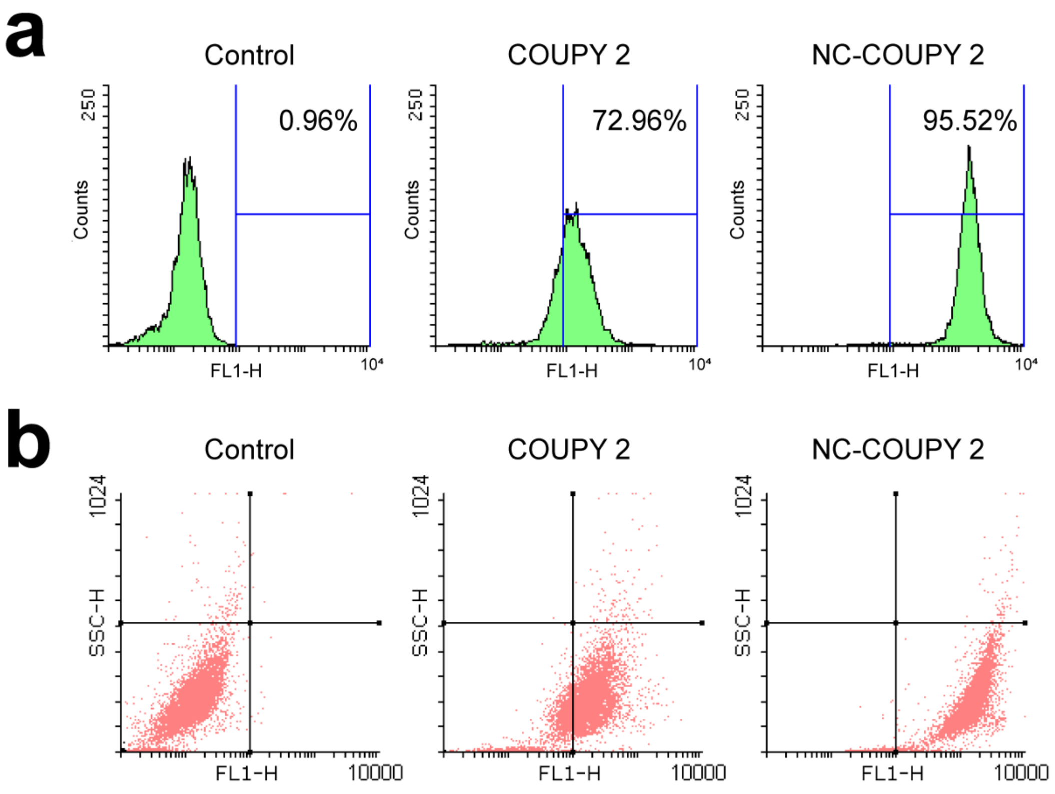


**Figure S23**. Flow cytometry analysis of ROS generation after phototreatments in HeLa cells. (**a**) Representative histograms of ROS generation as measured by DCF intensity (FL1-H) upon treatment with **COUPY 2** and **NC-COUPY 2** at 2 µM after irradiation (gated region corresponding to DCF+ cells). (**b**) Cell internal complexity (SSC-H) vs. DCF fluorescence (FL1-H) analysis of HeLa cells after phototreatment with **COUPY 2** and **NC-COUPY 2** at 2 µM.

### 8.4. Mitochondrial membrane potential assessment

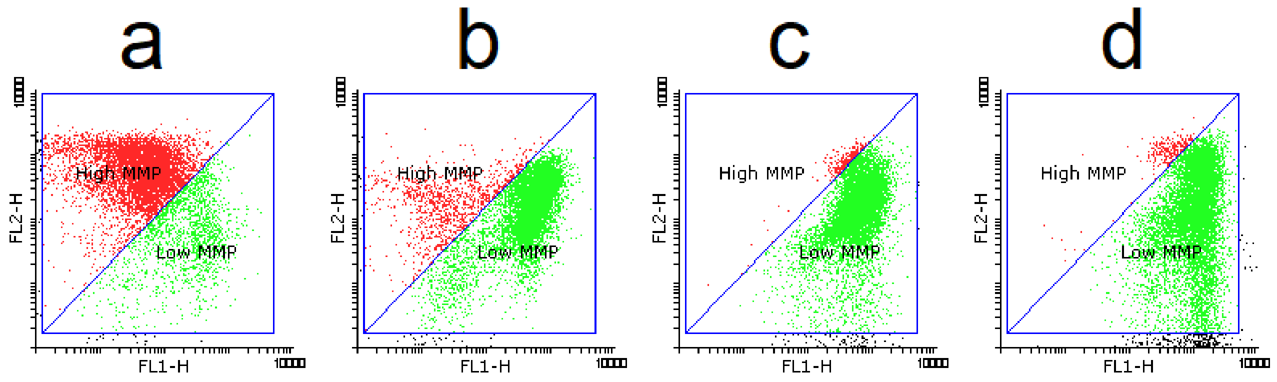


**Figure S24.** Evaluation of mitochondrial membrane potential (MMP). Representative flow cytometry dot plots of HeLa cells stained with JC-1 dye after visible light irradiation treatments with **COUPY 2** (c) or **NC-COUPY 2** (d) at $IC_{50}$ concentrations. Control cells (a) served as negative control, whereas 50 µM CCCP (b) was used as positive control. Green JC-1 monomers (low MMP) detected in FL1-H channel and red JC-1 aggregates (high MMP) in FL2-H ($\lambda_{exc}/\lambda_{em}$ = 488/530 and 620 nm respectively).

### 8.5. Apoptosis induction

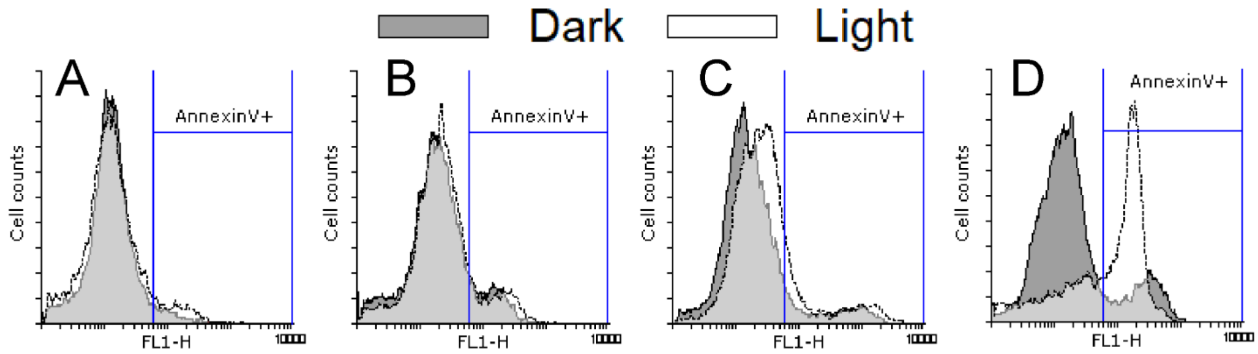


**Figure S25.** Representative flow cytometry histograms of HeLa cells stained with Annexin V-FITC after irradiation treatments with after visible light irradiation treatments with **COUPY 2** (C) or **NC-COUPY 2** (D) at $IC_{50}$ concentrations. Control cells (A) served as negative control, whereas 20 µM cisplatin (B) was used as positive control. Annexin V+ populations detected in FL1-H ($\lambda_{exc}/\lambda_{em}$ = 488/530 nm).

## 8.6. Autophagy detection

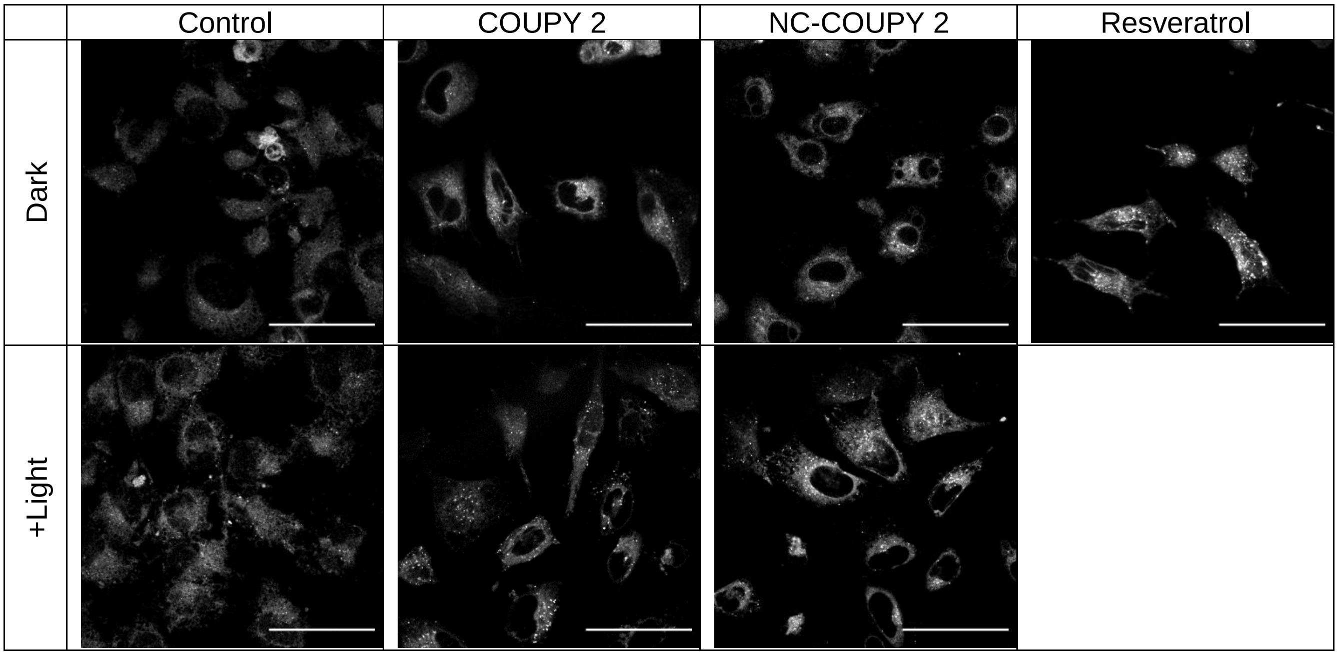


**Figure S26**. Autophagy detection in HeLa cells by confocal microscopy using monodansylcadaverine (MDC) staining after visible light irradiation treatments with **COUPY** 2 and **NC-COUPY 2** at $IC_{50}$ concentrations. Resveratrol (50 µM) was used as a positive control. Scale bar: 50 µm.

**8.7. Cell metabolism measurements.**

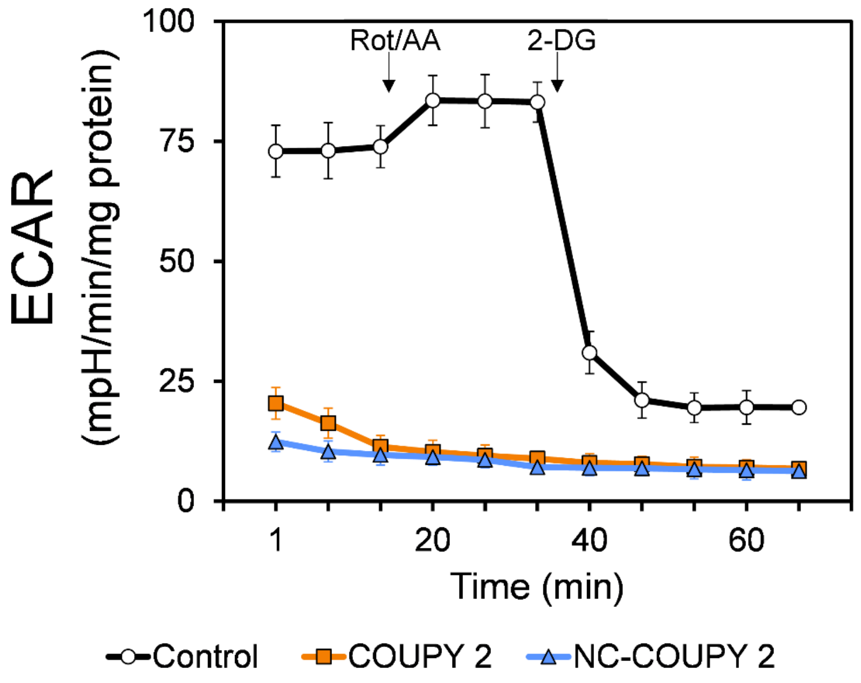


**Figure S27.** Glycolysis of HeLa cells as measured by extracellular acidification rate (ECAR) after 2 h treatment with tested compounds (10 µM). Data obtained with Seahorse XFe analyzer and represented as mean ± SEM (n= 4 replicates).

### 8.8. Cell cycle distribution

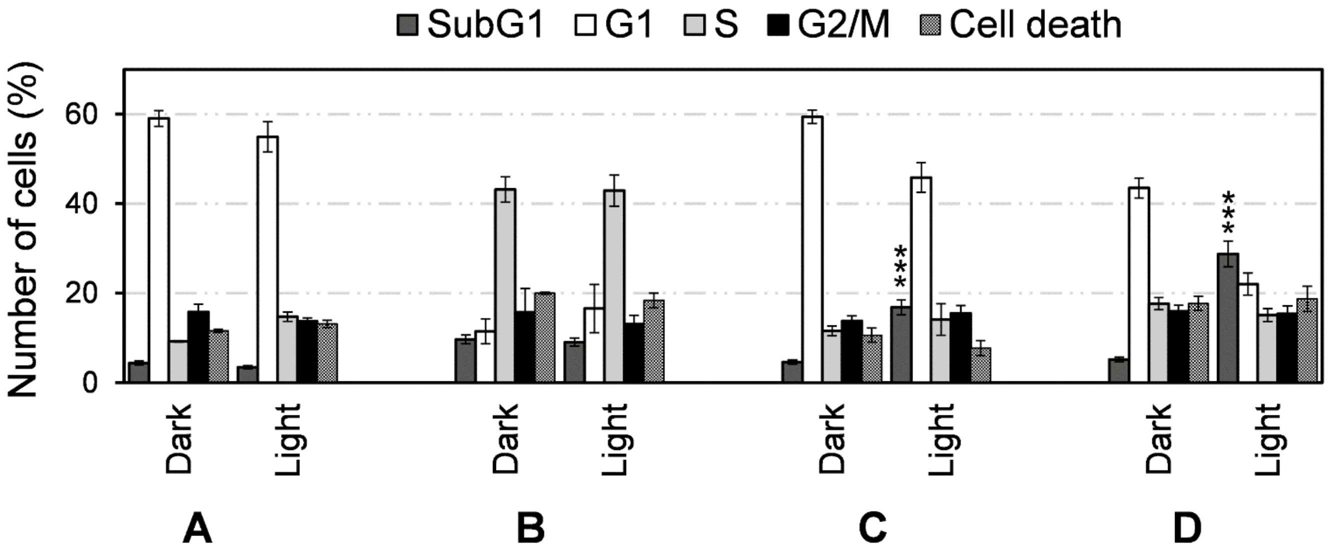


**Figure S28.** Cell cycle distribution analysis of HeLa cells as detected by propidium iodide staining after visible light irradiation treatments with **COUPY 2** (C) or **NC-COUPY 2** (D) at $IC_{50}$ concentrations. Control cells (A) served as negative control, whereas 20 µM cisplatin (B) was used as positive control. Data expressed as mean ± SD from three independent experiments. Statistical significant dark vs light from unpaired t-test (***p<0.001).

## 9. References

1.- a) A. Gandioso, R. Bresolí-Obach, A. Nin-Hill, M. Bosch, M. Palau, A Galindo, S Contreras, A Rovira, C Rovira, S Nonell, V Marchán. Redesigning the Coumarin Scaffold into Small Bright Fluorophores with Far-Red to Near-Infrared Emission and Large Stokes Shifts Useful for Cell Imaging. *J. Org. Chem*. **2018**, *83*, 1185–1195. b) E. Ortega-Forte, A. Rovira, A. Gandioso, J. Bonelli, M. Bosch, J. Ruiz, V. Marchán. COUPY Coumarins as Novel Mitochondria-Targeted Photodynamic Therapy Anticancer Agents. *J. Med. Chem*. **2021**, *64*, 17209–17220.
